# ENDOR characterization of an iron–alkene complex provides insight into a corresponding organometallic intermediate of nitrogenase†
†Electronic supplementary information (ESI) available: Additional spectroscopic and crystallographic information. CCDC 1543306. For ESI and crystallographic data in CIF or other electronic format see DOI: 10.1039/c7sc01602f



**DOI:** 10.1039/c7sc01602f

**Published:** 2017-06-30

**Authors:** Masaki Horitani, Katarzyna Grubel, Sean F. McWilliams, Bryan D. Stubbert, Brandon Q. Mercado, Ying Yu, Prabhuodeyara M. Gurubasavaraj, Nicholas S. Lees, Patrick L. Holland, Brian M. Hoffman

**Affiliations:** a Department of Chemistry , Northwestern University , Evanston , Illinois 60208 , USA . Email: bmh@northwestern.edu; b Department of Applied Biochemistry and Food Science , Saga University , Saga , 840-8502 , Japan; c Department of Chemistry , Yale University , New Haven , CT 06520 , USA . Email: patrick.holland@yale.edu; d Department of Chemistry , University of Rochester , Rochester , New York 14627 , USA

## Abstract

A bio-organometallic intermediate, denoted **PA**, was previously trapped during the reduction of propargyl alcohol to allyl alcohol (AA) by nitrogenase, and a similar one was trapped during acetylene reduction, representing foundational examples of alkene binding to a metal center in biology. ENDOR spectroscopy led to the conclusion that these intermediates have *η* ^2^ binding of the alkene, with the hydrogens on the terminal carbon structurally/magnetically equivalent and related by local mirror symmetry. However, our understanding of both the **PA** intermediate, and of the dependability of the ENDOR analysis on which this understanding was based, was constrained by the absence of reference iron–alkene complexes for EPR/ENDOR comparison. Here, we report an ENDOR study of the crystallographically characterized biomimetic iron(i) complex **1**, which exhibits *η* ^2^ coordination of styrene, thus connecting hyperfine and structural parameters of an Fe-bound alkene fragment for the first time. A tilt of the alkene plane of **1** from normal to the crystallographic Fe–C2–C1 plane causes substantial differences in the dipolar couplings of the two terminal vinylic protons. Comparison of the hyperfine couplings of **1** and **PA** confirms the proposed symmetry of **PA**, and that the *η* ^2^ interaction forms a scalene Fe–C–C triangle, rather than an isosceles triangle. This spectroscopic study of a structurally characterized complex thus shows the exceptional sensitivity of ENDOR spectroscopy to structural details, while enhancing our understanding of the geometry of a key nitrogenase adduct.

## Abstract 2

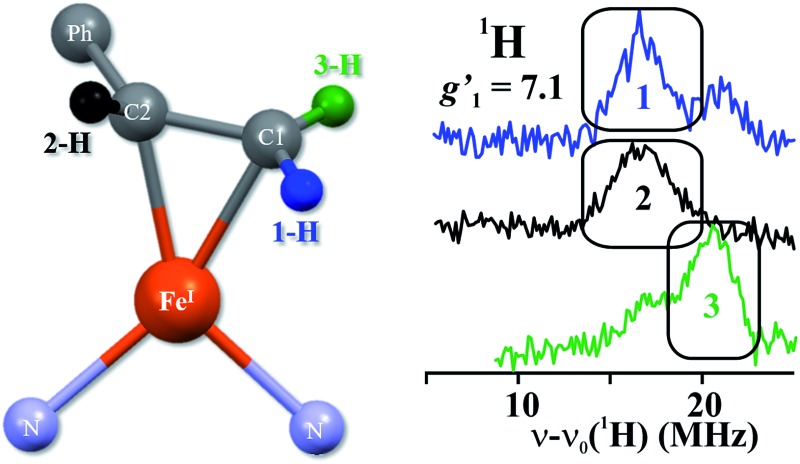
Comparison of an iron(I)–alkene complex to a nitrogenase intermediate using ENDOR reveals details of the binding geometry.

## Introduction

The binding of alkenes to transition metals is common in organometallic complexes,^1^ but in biological systems has rarely been observed. Copper-containing proteins bind ethylene in nature,^2^ and computations support the binding of acetylene to the metal in acetylene hydratase.^3^ A novel example of a bio-organometallic intermediate (denoted **PA**) was reported by one of us (B. M. H.), formed during the reduction of propargyl alcohol (**PA**) to allyl alcohol (AA) by the enzyme nitrogenase, with a similar state formed during the reduction of acetylene to ethylene.^4^ It is particularly interesting that this intermediate has Fe–C bonds to iron–sulfur clusters; since this report, there has also been spectroscopic evidence supporting ferraoxetane intermediates for catalytic [4Fe–4S] clusters.^5^ Most recently, ENDOR studies showed that a catalytically competent intermediate of radical-*S*-adenosylmethionine enzymes contains an Fe–C bond between a [4Fe–4S] cluster and the highly reactive 5′-deoxyadenosyl radical.^6^ The increased pace of discovery of compounds with metal–carbon bonds in biology further motivates the detailed spectroscopic characterization of biorelevant complexes with M–C interactions.

**Fig. 1:**
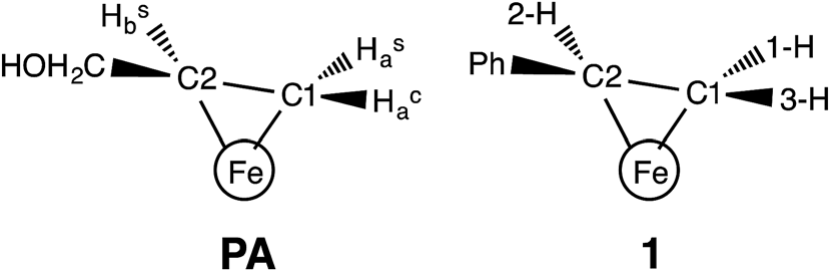
(Left) Proposed structure for bound AA in the **PA** intermediate of α-70^Ala^ nitrogenase. The carbon atoms have been renumbered from the original report^4^ to correspond to those of **1**. (Right) The core of **1**, which has styrene bound to a high-spin iron(i) center.

The aforementioned intermediate in nitrogenase was shown to contain AA as a complex in which the double bond of AA undergoes *η* ^2^ binding to a single Fe of the iron–molybdenum catalytic cofactor (FeMo-co) of nitrogenase (Fig. 1 (left)). This interpretation was later supported by EXAFS and NRVS studies of **PA**.^7^ This complex, in which the iron and two carbon atoms form a three-membered ring, can be described as a π-alkene or as a ferracyclopropane according to the Dewar–Chatt–Duncanson model.^1^

In our earlier work, a series of ^1^H/^2^H and ^13^C ENDOR experiments showed that the nitrogenase **PA** intermediate contains an adduct of AA.^4^ The binding site was assigned to the Fe 2, 3, 6, 7 face of FeMo-co, in part because **PA** is reduced only upon mutation of the α-70 residue from Val to Ala to create space for the large substrate. The bound species was assigned as the alkene product of alkyne reduction, rather than a singly-reduced intermediate, because isotopic labeling studies showed that two H atoms from solvent had been added to the alkyne reactant. The binding geometry (Fig. 1 (left)) was inferred from the surprisingly identical hyperfine parameters for the two H atoms (Hsa and Hca) on the terminal carbon. However, the coordination chemistry precedents for this proposal were relatively distant Os and Ta complexes, because there were no literature examples of EPR-active, structurally characterized iron–alkene complexes for comparison. Thus, an Fe–alkene complex that is both crystallographically characterized and paramagnetic would enable a comparison of the ENDOR parameters of a precisely known structure with those of the FeMo-co–allyl alcohol complex, thereby enhancing our understanding of the intriguing **PA** intermediate.

Here, we provide a detailed analysis of the iron(i) complex described below, L^Me^Fe(styrene) (L^Me^ = 2,4-bis(2,6-diisopropylphenylimido)-3-pentyl) (**1**; Fig. 1 (right)), which one of us (P. L. H.) reported earlier.^8^ This compound has a high-spin *S* = 3/2 ground state, and its *η* ^2^ binding is midway between the π-alkene and ferracyclopropane resonance structures, as shown by crystallography and NMR spectroscopy. Strong backbonding was supported by computations for an analogous *η* ^2^-alkyne complex.^9^ Most importantly, **1** has a monosubstituted alkene with three protons in the same *η* ^2^ disposition as the FeMo-co-bound **PA** intermediate with bound allyl alcohol. Here, we show ENDOR studies of compound **1** and isotopologues that demonstrate the sensitivity of ENDOR to subtle structural features of such a complex, and give additional insight into the geometrical details of the **PA** intermediate.

## Materials and methods

### Sample preparation

Samples were synthesized, purified, and introduced into sample tubes in an MBraun glovebox in an N_2_ atmosphere maintained at or below 1 ppm of O_2_. Glassware and sample tubes were dried at 150 °C for at least 12 hours before use. Pentane, diethyl ether, benzene, and toluene were purified by passage through activated alumina and Q5 columns (Glass Contour Co., Laguna Beach, CA). All solvents were stored over activated 3 Å molecular sieves and passed through a plug of activated alumina before use. Deuterated cyclohexane was dried over potassium/benzophenone and vacuum transferred to a storage container before use. Graphite, Celite, and 3 Å molecular sieves were dried at 300 °C under vacuum for >12 h. Styrene-d_3_ and styrene-d_5_ were obtained from ICON Isotopes and vacuum distilled prior to use. Styrene-d_1_ and styrene-d_2_ were synthesized through published methods.^10^ α-^13^C-styrene and β-^13^C-styrene were obtained from Sigma-Aldrich (>98% ^13^C) purified by the freeze–pump–thaw degassing method. The iron complex **1** and its isotopologues were prepared according to the literature procedure, and characterized as described.^8^ ^1^H NMR spectra of the isotopologues are shown in the ESI.† Samples for ENDOR spectroscopy were prepared by dissolving iron complexes in toluene : benzene (3 : 1) at a concentration of 2.1–2.5 mM in a quartz tube, and flash-freezing the solutions to 77 K.

### Crystallography of **1**

Low-temperature diffraction data (ω-scans) were collected on a Rigaku SCX Mini diffractometer coupled to a Rigaku Mercury275R CCD with Mo Kα radiation (*λ* = 0.71073 Å). The diffraction images were processed and scaled using the Rigaku CrystalClear software.^11^ The data were solved with SHELXT and were refined against *F* ^2^ on all data by full-matrix least squares with SHELXL.^12^ All non-hydrogen atoms were refined anisotropically. Unless stated otherwise, all hydrogen atoms were included in the model at geometrically calculated positions and refined using a riding model. (DFT was used to determine their positions for comparison to spectroscopic measurements, as described below.) The isotropic displacement parameters of all hydrogen atoms were fixed to 1.2 times the *U* value of the atoms to which they are linked (1.5 times for methyl groups). The terminal hydrogen atoms of the vinyl group in styrene were found in the difference map and freely refined. However, their atomic positions converged at chemically unreasonable values. Ultimately, geometrically restrained, riding atoms were used for H24 and H28. One of the two hexane solvent molecule was disordered near the crystallographic inversion center. The two positions were modeled with atoms {C10, C20, C30} as the major component set, where {C40, C50, C60} is the minor set. Numerous restraints were needed to maintain a chemically reasonable structure for the hexane solvent molecules. The site occupancies were freely refined and fixed at their converged values of 0.74(1) and 0.26(1), respectively. The full numbering scheme of complex **1** can be found in the ESI.† Full details of the X-ray structure determination are in the ESI.†

### EPR and ENDOR spectroscopy

X-band CW EPR measurements were performed on a Bruker ESP 300 spectrometer equipped with an Oxford Instruments ESR 910 continuous He flow cryostat. Q-band CW EPR and ENDOR spectra were collected at 2 K on a spectrometer with a helium immersion Dewar as previously reported.^13^ Stochastic field-modulation detected ENDOR technique, first reported by Brueggeman and Niklas,^14^ was utilized for ^1^H ENDOR measurement. In the stochastic ENDOR sequence, the RF is randomly hopped over the frequency range of the spectrum with the subtraction of a background signal (RF off) at each frequency. Pulsed ENDOR spectra were collected on a spectrometer described earlier,^15^ equipped with a helium immersion Dewar for measurements at a temperature of 2 K. ENDOR measurements employed the Mims pulse sequence (π/2–*τ*–π/2–*T*–π/2–*τ*-echo, RF applied during interval *T*).^16^

For a nucleus with hyperfine coupling, *A*, Mims pulsed ENDOR has a response *R* which depends on the product, *Aτ*, according to eqn (1),

1 *R* ∼ [1 – cos(2π*Aτ*)]

This function has zeros corresponding to minima in the ENDOR response (hyperfine “suppression holes”), at *Aτ* = *n*, with *n* = 0, 1,…, and maxima at *Aτ* = (2*n*+1)/2 with *n* = 0, 1,….^17^ The “holes” at *A* = *n*/*τ* with *n* = 1, 2, 3… can be adjusted by varying *τ*. However, the central *n* = 0 hole at *ν* = *ν*_N_ persists. This can be of significance in distinguishing a tensor that is dominated by anisotropic interactions from one that is dominated by isotropic ones. The latter would never lead to ENDOR intensity near *ν*_N_; the former does so for certain orientations, but the *ν* = 0 Mims hole tends to diminish the differences between the two cases.

### Density-functional theory calculation for hyperfine coupling constants of **1**

DFT calculations used the ORCA program package, version 3.0.3.^18^ Calculations reported here employed the B3LYP functional.^19^ We used the Ahlrichs triple-ζ-quality basis set with one set of polarization functions, def2-TZVP,^20^ with ZORA relativistic corrections.^21^ SCF convergence was accelerated with the RI approximation using the def2-TZVP/J auxiliary basis set. An empirical van der Waals correction was applied to the DFT energy (D3BJ).^22^ The SCF calculations were tightly converged (TightSCF) with unrestricted spin (UKS). Geometry optimization used TightOpt criteria, and a numerical frequency calculation on the optimized geometry showed no negative frequencies. Hyperfine coupling constants were calculated in ORCA using this geometry but substituting the EPR-II basis set on C and H atoms.^23^

## Results

### Structure of compound **1**

**Fig. 2:**
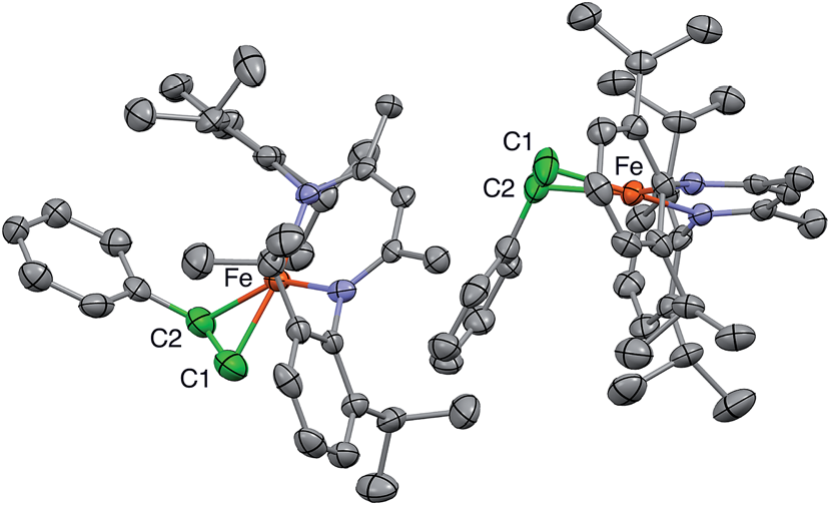
Plot of 50% probability ellipsoids depicting the X-ray crystallographic structure of **1**, which consists of two independent molecules. Hydrogen atoms and two co-crystallized solvent molecules are omitted for clarity. The alkene carbons are colored green to orient the reader.

The structure previously reported^8^ for **1** has anomalies that suggest unmodeled disorder in the core of the molecule. Re-refinement of the previous diffraction data showed that the best model for the published data has two positions of the Fe–diketiminate unit in a 1 : 1 ratio, and two positions of the styrene in a 4 : 1 ratio, in which the C=C bonds are at right angles to one another. This physically unreasonable model is unsuitable for correlation with the ENDOR-derived parameters. Fortunately, we were able to grow a different crystal of a hexane solvate that gives a more reasonable model, with two molecules in the asymmetric unit (Fig. 2).

**Fig. 3:**
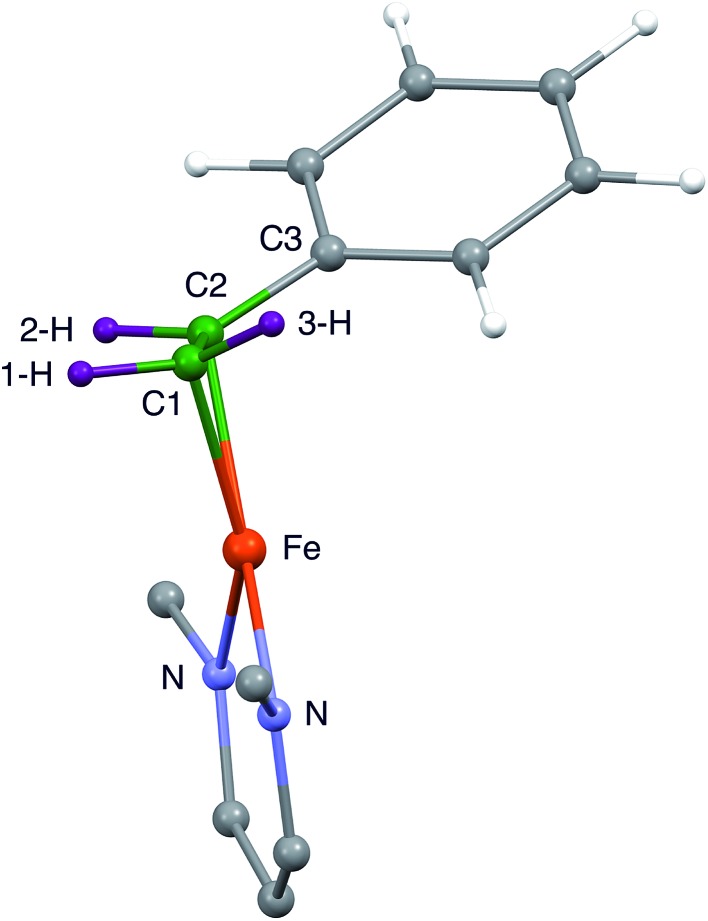
Side view of the core of the DFT model of **1**, showing that the phenyl group is displaced away from the diketiminate supporting ligand. This tilts the plane of the alkene and also places C2 further than C1 from Fe.

In the new structure, the two independent molecules have very similar core structures (Table 1), each having the C=C double bond very close to the iron–diketiminate plane. The C=C bond lengths are equivalent at 1.384(8) and 1.380(8) Å, a distance that is significantly longer than that in free styrene (1.32 Å).^24^ This supports the description of this double bond as a π-complex with significant backbonding from the iron(i) ion into the π* orbitals of the alkene. Though one might imagine that the face-on binding mode of the alkene would make the C1–C2–C3 plane perpendicular to the Fe–C1–C2 plane (*i.e.* dihedral angle of 90° between the alkene plane and the iron–alkene plane), the phenyl substituent is tilted away from the metal to give a dihedral angle of 74 ± 1° between the C1–C2–C3 and Fe–C1–C2 planes. The distortion is presumably caused by steric interactions between the phenyl group and the supporting ligand, which twists the styrene around the axis formed by the C–C double bond (Fig. 3). The positions of the hydrogen atoms were not accurately obtained from the crystallographic analysis, because the electron density peaks for hydrogen atoms are small and diffuse from X-ray crystallography.

**Table 1:** Crystallographic and calculated structural parameters for **1**

	Crystal molecule 1	Crystal molecule 2	DFT optimization
Fe–N	1.955, 1.977	1.963, 1.979	1.987, 1.993
Fe–C1	1.996	2.015	2.001
Fe–C2	2.020	2.033	2.062
C–Fe–C	40.2	39.4	41.0
Dihedral (C–Fe–C to N–Fe–N)	9.9	6.3	20.2
Dihedral (C–Fe–C to C–C–C)	74.8	74.1	61.2
Fe–1-H			2.568
Fe–2-H			2.604
Fe–3-H			2.632
CCC plane – 1-H			0.460
CCC plane – 2-H			0.416
CCC plane – 3-H			0.009

It is notable that the phenyl-bearing carbon C2 is further from the Fe atom than C1 by 0.020(7) Å in both independent molecules. Thus, the phenyl group on the alkene in **1** has two major influences on the geometry of alkene binding. (i) The Fe–C1–C2 triangle of **1** is scalene, not isosceles, with a longer distance to C2. (ii) The C1–C2–C3 plane tilts to place the phenyl group (and presumably the 3-H atom which is *cis* to the phenyl) further from the Fe atom.

Because the crystallographic analysis did not give the desired level of detail on the locations of the hydrogen atoms, we used density-functional calculations (B3LYP/def2-TZVP) to optimize a model of **1** with all atoms included. Fig. 3 shows the core of the optimized model, with the carbon and hydrogen atoms near the Fe colored green and purple, respectively, to highlight their positions. The DFT-optimized metrical parameters were in good agreement with the crystallographically determined structure (Table 1). There are some slight differences in the dihedral angles, which may result from the lack of crystal packing effects in the geometry optimized structure. The DFT model reproduces the crystallographically observed tilting of the phenyl group away from the iron as well as the difference between the two Fe–C bond distances. Importantly, the DFT model shows the hydrogen atom positions, and as expected the “tilt” of the alkene plane results in 3-H (*cis* to the phenyl) being furthest from the Fe center (see Table 1).

### EPR spectra

**Fig. 4:**
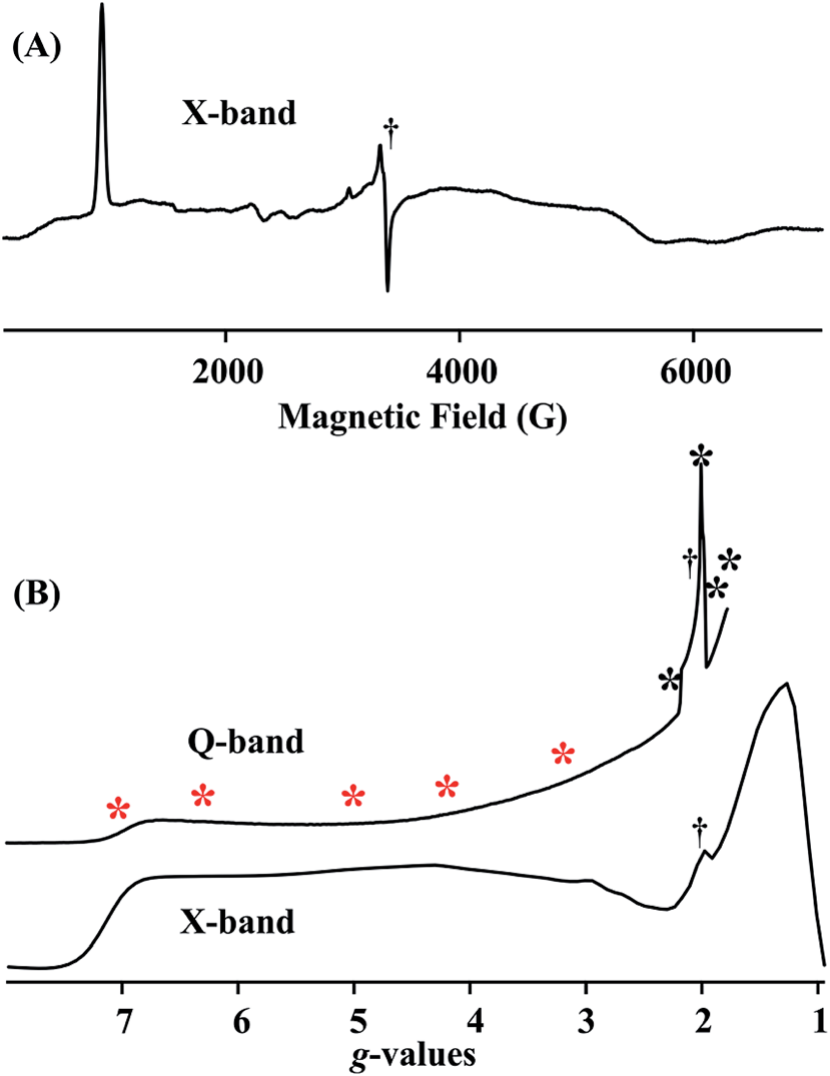
(A) X-band CW EPR spectrum of **1**. Conditions: *T* = 9 K, frequency = 9.373 GHz, microwave power = 10 mW, 100 kHz modulation amplitude = 10 G. (B) Q-band CW EPR rapid-passage spectrum and X-band *g*-scale absorption-display EPR spectrum generated by integration of spectrum in (A). Conditions for Q-band EPR: *T* = 2 K, frequency = 35.28 GHz, microwave power = 1 mW, 100 kHz modulation amplitude = 1.3 G. Asterisks show the ENDOR collection fields; red = CW stochastic/rf-sweep ^1^H; black = pulsed Mims ^2^H (†: impurity signals).

Fig. 4A and B (lower) show the derivative and absorption display X-band CW EPR spectrum of the *S* = 3/2 complex **1**, whose highly anisotropic signal can be described in terms of a center with fictitious *S*′ = 1/2 spin that exhibits an effective *g*-tensor, ***g*** ′ = [7.1, 1.3, 1.1]. This can be explained in terms of the transitions within the low-lying Kramers doublet of a true *S* = 3/2 (d^7^) system^25^ that exhibits a large and rhombic zero field splitting: |*D*| ≫ 0.3 cm^–1^, *D* < 0; rhombicity parameter, *λ* = |*E*/*D*| = 0.22; and intrinsic *g*-values for the true *S* = 3/2 spin state, ***g*** = [2.48, 2.00, 1.91]. The EPR spectrum also exhibits minor contributions at around *g* ∼4, ∼3 and ∼2, most likely from small amounts of decomposition and/or oxidized impurity. Fig. 4B (upper) displays the portion of the 2 K Q-band EPR absorption spectrum for compound **1** up to the highest magnetic field available. The asterisks in Fig. 4B show the fields at which ENDOR measurements were performed.

### ^1^H/^2^H ENDOR spectra

**Fig. 5:**
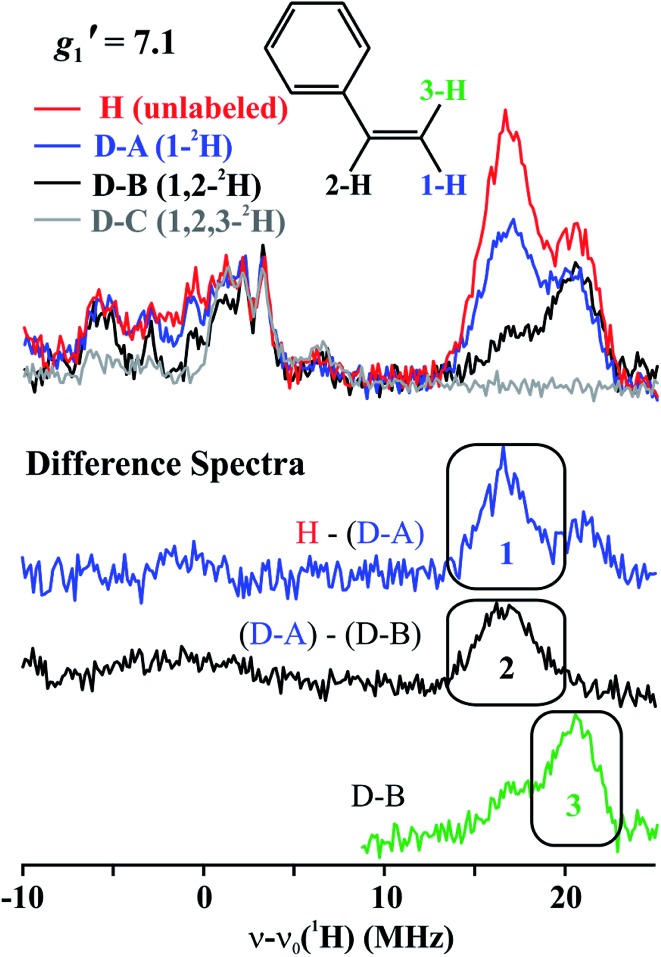
(Top) *g*′_1_ = 7.1 Q-band CW stochastic ^1^H ENDOR spectra of **1** with unlabeled and ^2^H labeled styrene, as indicated. Conditions: *T* = 2 K, microwave frequency = ∼35.2 GHz, microwave power = 1 mW, 100 kHz modulation amplitude = 1.3 G, rf is random hopping in the cycle; rf-on = 2 ms, rf-off = 2 ms, sample collection time = 1 ms. (Bottom) Difference ENDOR spectra, as indicated.

The top of Fig. 5 presents Q-band CW stochastic single-crystal-like ^1^H ENDOR spectra collected at *g*′_1_ = 7.1 from **1** prepared with the styrene H/D isotopologues illustrated in Fig. 5: H (unlabeled), D-A (1-^2^H), D-B (1,2-^2^H) and D-C (1,2,3-^2^H). The spectra show a strongly-coupled ^1^H *ν*_+_ pattern whose features are offset from the ^1^H Larmor frequency by one-half of their apparent hyperfine coupling, *A*′/2 = (*g*′_1_/*g*_e_)*A*/2 ∼ 17 MHz, which corresponds to an intrinsic hyperfine coupling to the *S* = 3/2 ion of *A* ∼10 MHz. This pattern contains contributions from all three vinyl protons as shown by the changes as the three protons of styrene (sample H) are replaced by deuterons in one (D-A), two (D-B) and all three (D-C) sites (Fig. 5, bottom). The feature contains no contributions from the aryl protons, as it disappears in the D-C spectrum, where all three vinyl protons are replaced with deuterium.

The contribution from the 1-H proton is seen in the difference spectrum [H–(D-A)]; that of the 2-H proton likewise is provided by the [(D-A) – (D-B)] subtraction; that of 3-H is seen directly in the D-B spectrum. We assign the subsidiary signals of 1-^1^H and 3-^1^H to minor contamination (estimated as less than 30%) with unexchanged 3-H and 1-H and/or 2-H. From the frequencies of the peaks one obtains *A*′_1_(1-^1^H) ∼ *A*′_1_(2-^1^H) ∼33 MHz and *A*′_1_(3-^1^H) ∼42 MHz. Thus, these single-crystal-like spectra immediately reveal that the terminal hydrogens, (1-^1^H) and (3-^1^H), are not magnetically equivalent, indicative of deviations at the C1 terminal alkene carbon from idealized mirror symmetry through the Fe–C–C bonded triangle, in contrast to the identical couplings for the terminal H atoms and mirror symmetry for **PA**.

**Fig. 6:**
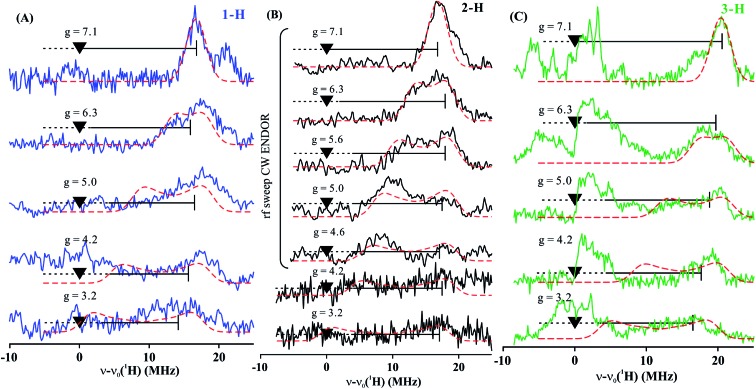
Two-dimensional frequency pattern of ^1^H CW stochastic/rf sweep ENDOR spectra for (A) 1-H, (B) 2-H, and (C) 3-H. Dashed red lines represent simulations with the GENDOR program^26*c*^ using *A* and *g*′ tensors given in Table 2.

To estimate the full hyperfine coupling tensors^26^ and their orientations for the vinyl protons, a 2D field-frequency pattern of ^1^H CW stochastic/rf sweep ENDOR spectra was collected over the field range from *g*′_1_ to *g* ∼ 3; at higher fields (Fig. S1†) the extreme anisotropy of the effective *g*′ tensor of the *S* = 3/2 Fe(i) ion of **1** degrades both the intensity and the resolution of the ^1^H spectra. Although Mims ^2^H ENDOR signals for **1** could be collected at the higher fields (Fig. S2†), they were only of minimal use, as they were poorly resolved and distorted by Mims ‘holes’.^27^ We generated 2D patterns for the individual 1-H and 2-H vinyl protons, shown in Fig. 6 from the CW spectra collected at *g* > 3 by subtracting the sets of spectra as for Fig. 5; the pattern for 3-H in Fig. 6C was obtained directly from **1** with D-B (1,2-^2^H).

The experimental 2D ^1^H/^2^H ENDOR patterns do not cover enough of the broad field range for this highly anisotropic *S* = 3/2 signal to achieve a unique fit to the ^1^H/^2^H hyperfine tensors, so we used the DFT model of **1** (see above) to calculate the intrinsic *S* = 3/2 hyperfine tensors for the vinyl protons and carbons of **1** (Table 2). The peak positions in the spectra taken at the single-crystal-like *g*′_1_ field value are well reproduced with the orientations of the tensors relative to the *g* tensor, to which the simulations are extremely sensitive. These values are listed in Table 2. Note that the DFT computations are not expected to give an accurate *g* tensor. Spectra at multiple fields were simulated using the calculated tensor components and fit orientations, and the resulting simulations are overlaid on the experimental 2D pattern in Fig. 6. Overall, this simulation procedure satisfactorily reproduces the experimental 2D pattern, supporting the synergistic use of ENDOR measurements and DFT computations to determine the proton hyperfine tensors of this high-spin iron(i) ion.

**Table 2:** DFT-derived intrinsic *S* = 3/2 hyperfine tensors for vinyl protons and carbons of the styrene ligand in **1**, compared to experimentally-derived hyperfine tensors for the nitrogenase **PA** intermediate

		*A*_1_, *A*_2_, *A*_3_ (MHz)	*A*_iso_ (MHz)	*β* ^*a*^ (°)
**1** (styrene)^*a*^	1-H	13.9, –0.69, 0.89	+4.7	41
	2-H	14.3, 2.4, –0.27	+5.5	41
	3-H	16.1, 4.7, 3.0	+8.0	41
	C1	–12.8, –7.4, –5.2	–8.5	ND
	C2	–12.5, –4.9, –3.0	–6.8	—
Nitrogenase **PA** intermediate^*b*^	H_a_[c,s]	3, 16, 22.5	13.8	
	H_b_	—	∼4	
	C1	5.1, 2.4, 3.5	3.7	
	C2	0.55, 1.3, 1.4	1.1	

^*a*^Hyperfine tensor components calculated by DFT; for ^1^H, values are supported by simulations using the Euler angle relating ***A*** to ***g*** derived from ENDOR simulation (see text). The uncertainty of the angles is ±1°.

^*b*^From ref. 4; relative signs are experimentally determined, not absolute signs.

In next making comparisons between the couplings for alkenes bound to **1** and **PA**, we take into account that the measured couplings for each nucleus of the alkene bound to the Fe of the **PA** intermediate (*A*) are modified from those for the alkene bound to an isolated Fe ion (*A* ^int^), such as **1**, because the electron spins of the cluster Fe ions are coupled to give an total cluster spin of *S* = 1/2.^28^ As a result of this coupling the hyperfine interaction measured by ENDOR for each nucleus of the alkene bound to an Fe of the **PA** intermediate (*A*) is modified from that for the isolated Fe ion (*A* ^int^) through multiplication by a spin-coupling coefficient that is determined by nature of the spin coupling within the cluster, and may be greater or less than unity.

The principal components of the proton hyperfine interactions are the sum of an isotropic term, reflecting spin delocalization into the proton 1s orbital, and a traceless dipolar interaction with nearby spin density, *A*_i_ = *A*_iso_ + *T*_*i*_ (where *i* = 1–3). The *T*_*i*_ values are dominated by interaction with the spin on the bonded Fe, and vary inversely with the cube of the Fe–H distance. For comparisons, the dipolar coupling can be exemplified by the principal component of largest absolute value, *T*_max_. The different values of *T*_max_ for the terminal vinylic protons of **1**, with that for 1-H (9.2 MHz) being larger than that for 3-H (8.1 MHz), reflect the different calculated distances of these protons from Fe (2.568 Å *vs.* 2.632 Å, Table 1) caused by the tilt of the styrene. Correspondingly, *T*_max_ for 2-H of **1** (8.8 MHz) lies between those for 1-H and 3-H, as does its distance from Fe (2.604 Å). These differences reflect the distorted geometry of **1**, and contrast with the identical couplings for the terminal vinylic protons of **PA**.

The value of *T*_max_ = 10.8 MHz for the two identical terminal protons of **PA** is reasonably similar to the values for the three vinylic protons of the styrene of **1** (8.1 to 9.2 MHz, Table 2). Likewise, the isotropic coupling, *A*_iso_, for the terminal vinyl protons of the **PA** organometallic intermediate in nitrogenase, Table 2, is of similar magnitude to the average for those for the vinyl protons of **1**, as shown in Table 2. Though the values are close, both *T*_max_ and *A*_iso_ are somewhat greater for **PA** than **1**, suggesting that the vector coupling coefficient of the alkene-coordinated Fe ion of **PA** may be greater than unity.

### ^13^C ENDOR spectra

To further characterize **1**, we collected CW ENDOR spectra for **1** with individual ^13^C labels at the two vinyl carbons. Fig. S4† displays ENDOR spectra at *g*′_1_ for ^13^C1 labeled (red) and unlabeled (gray) **1**, and their difference spectrum (black). The spectrum at this field exhibits ENDOR signals from ^13^C, weakly coupled ^1^H and strongly coupled ^14^N responses from the iron ligand of the diketiminate. As indicated in the figure, the difference spectrum at the *g*′_1_ single-crystal-like field shows a broad signal from ^13^C1 that arises from doublets split by the twice the ^13^C Larmor frequency and centered over a range of effective hyperfine coupling, *A*(^13^C)′/2 ∼ 10–15 MHz, corresponding to distributed intrinsic couplings *A*(^13^C)/2 = [*A*(^13^C)′/2] × (*g*_e_/*g*′_1_) ∼2.8–4.2 MHz. In the context of the DFT calculations, this suggests that the hyperfine tensor may be substantially rotated away from *g*′_1_. Unfortunately, the ^13^C difference signal (as well as the ^14^N signal) is lost as the field is raised, precluding any experimental determination of the full ^13^C1 hyperfine tensor.

We also collected CW ENDOR spectra at *g*′_1_ for ^13^C2 labeled **1**. However, the difference spectrum with unlabeled **1** failed to show any unambiguous ^13^C responses at *g*′_1_ or higher fields. We suggest this may reflect an even broader distribution in hyperfine couplings.

## Discussion

This study shows that the ^1^H hyperfine couplings to the three vinyl protons of **1** reliably report the bonding and geometry of the alkene in this iron(i) complex, and they thus can be profitably compared to those of the AA protons of **PA**. Even though a number of factors reduce the precision for the vinyl ^1^H hyperfine couplings of **1** relative to those observed for the **PA** intermediate, comparison of the two systems yields structural information that was not previously available in the absence of the model complex.

**Fig. 7:**
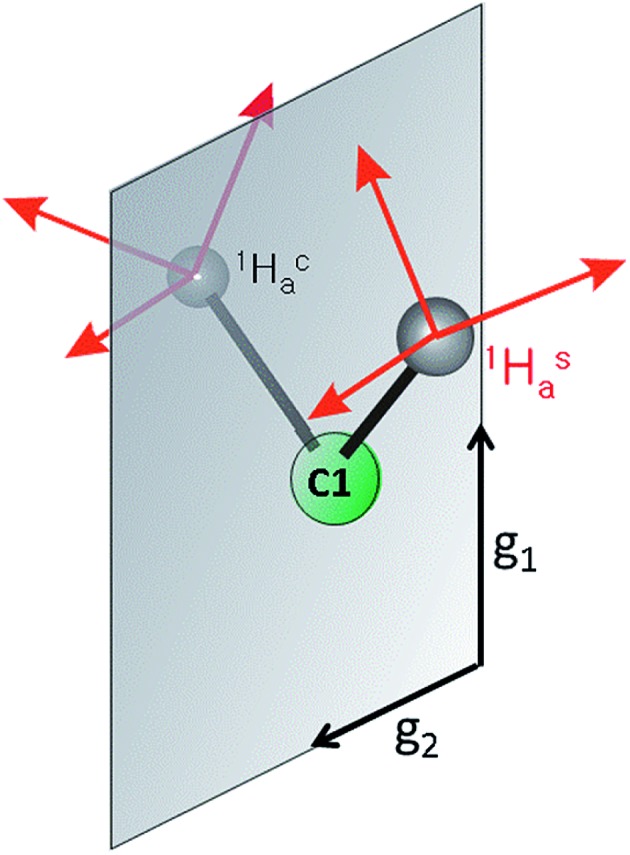
Mirror plane relating Hsa and Hca in **PA**.

The key element in assigning the *η* ^2^ structure of **PA** (Fig. 1) was the identical hyperfine coupling tensors for the two terminal ^1^H of the cluster-bound AA, one of which originated in the **PA** substrate (Hca) and one from solvent (Has) (Fig. 7).^4^ The *S* = 1/2 spin state of the **PA** intermediate, with its narrow *g*-spread, ***g*** = [2.123, 1.998, 1.986], allowed the 2D pattern of ENDOR spectra to be collected with high resolution across the entire EPR envelope of **PA**, and the ENDOR spectra from the two ^1^H overlay precisely at each field within the envelope. Likewise, the higher resolution of ^2^H spectra again yielded identical spectra for ^2^Hsa and ^2^Hca. These observations showed that Hca and Hsa exhibit magnetic equivalence, which in turn implies that these hydrogens are structurally equivalent and related by a local mirror symmetry, as illustrated by Fig. 7.

Analysis of the *η* ^2^-alkene reference structures in the Cambridge Database at that time (M = Ta and Os, but not Fe) indicated that the only way for the vinylic protons of a bound alkene to be related in this way was through the π-alkene/metallacyclopropane structure.^4^ Specifically, these structures have high local symmetry at C1 that is consistent with the ENDOR measurements for the AA bound to FeMo-co in **PA**. In contrast, a binding mode in which the vinyl carbon atoms (C1 and C2) of the AA product each bind to a different Fe ion (dimetallacyclobutane) would be nonplanar (as in a known diosmium complex^29^), rendering the two C1 protons stereoelectronically and magnetically inequivalent.

Whereas the signals for Hca and Has of **PA** overlay at all fields, the spectra of **1** collected at the single-crystal-like field of *g*′_1_ = 7.1 (Fig. 5) show small but clear differences in the coupling to 1-H and 3-H. The largest ^1^H dipolar couplings (*T*_max_) are a good marker for the through-space distance from the Fe ion to the proton, and these values differ significantly for the terminal 1-H and 3-H of **1** (9.2 *vs.* 8.1 MHz, Table 2) as a consequence of the differing distances from Fe caused by the tilt of the alkene plane. Thus, the observation of inequivalent hyperfine couplings for the distorted structure of **1** supports the previous conclusion that a local mirror plane through the Fe–C–C unit is needed to observe equivalent couplings in **PA**. It is likely that the smaller size of the –CH_2_OH substituent of AA allows a more symmetric ‘face-on’ binding with no tilt, whereas the bulkier and more rigid phenyl substituent in **1** tilts the CH_2_ plane. Overall, it is evident that ^1^H ENDOR spectroscopy is an exceptionally sensitive reporter of distortions of the bound alkene.

The sensitivity of the ENDOR parameters of the two terminal ^1^H of a bound alkene to the small tilt in **1** supports the earlier rejection of a structural model in which the vinyl carbons (C1 and C2) of the AA each bind to a different Fe ion, because an alkene as part of a dimetallacyclobutane ring would be nonplanar, with an even larger tilt of the C1-Hca/Hsa fragment of bound AA. In short, the comparison of **PA** with **1** further substantiates the *η* ^2^ binding in **PA** shown in Fig. 1A, with the C2–C1 (Hca/Hsa) plane of the AA alkene product perpendicular to the Fe–C2–C1 plane.

Additional key features of the **PA** analysis were that the C2 vinyl proton of **PA** (H_b_) has a distinctly smaller isotropic coupling than the two H_a_, and that C2 of AA has a much smaller hyperfine coupling than C3, which together were interpreted as implying that the Fe–C–C triangle is not equilateral, but scalene, with a longer bond to C2.^4^ The crystal structure of **1** shows a distortion in this direction, although with a difference in distances of only 0.02 Å. Correspondingly, although the estimated isotropic couplings for the C1 protons (1-H, 3-H) differ because of the structural distortion, their average is quite close to that for the C2 proton (2-H), again consistent with the small difference in Fe–C bond lengths. Thus, the data on the model complex support the conclusion that the Fe–C–C triangle in the enzyme environment is more distorted.

## Conclusions

The ENDOR characterization of the crystallographically characterized biomimetic Fe complex **1**, with its *η* ^2^ coordination of styrene, provides an experimental connection between hyperfine and structural parameters of an alkene fragment on iron for the first time. The small tilt of the alkene plane in **1** away from normal to the Fe–C2–C1 plane causes a significant difference in the dipolar couplings of the two terminal vinylic H. This shows that the ENDOR measurements are highly sensitive to such structural details. The different couplings in the model contrast sharply with the identical couplings for the two terminal vinylic protons of AA in the **PA** intermediate, and this study thus shows that the AA vinylic fragment indeed binds face-on to Fe with negligible tilt of the alkene plane. In addition, the similar isotropic couplings of the protons on C1 and C2 of **1** correlate with a small difference in the Fe–C distances. The larger differences between the protons on C1 and C2 in the **PA** intermediate support the idea that the Fe–C distances to the alkene carbons of the **PA** nitrogenase intermediate differ significantly more than is observed for **1**.
