# Supplementary material for: ENDOR characterization of an iron–alkene complex provides insight into a corresponding organometallic intermediate of nitrogenase
Source: Chem Sci. 2017 Jun 30;8(9):5941–8. doi: 10.1039/c7sc01602f (PMC5620524; doi:10.1039/c7sc01602f)
Supplement: Supplementary file 1 [file SC-008-C7SC01602F-s001.pdf]

Electronic Supplementary Material (ESI) for Chemical Science.
This journal is © The Royal Society of Chemistry 2017

Supporting Information for:

ENDOR Characterization of an Iron-Alkene Complex Provides Insight into a Corresponding Organometallic Intermediate of Nitrogenase

Masaki Horitani[¶], Katarzyna Grubel[†], Sean F. McWilliams[†], Bryan D. Stubbert[‡], Brandon Q. Mercado[†], Ying Yu[‡], Prabhuodeyara M. Gurubasavaraj[‡,+], Nicholas S. Lees[¶], Patrick L. Holland[†,], Brian M. Hoffman[¶,]**

*e-mail: patrick.holland@yale.edu; bmh@northwestern.edu

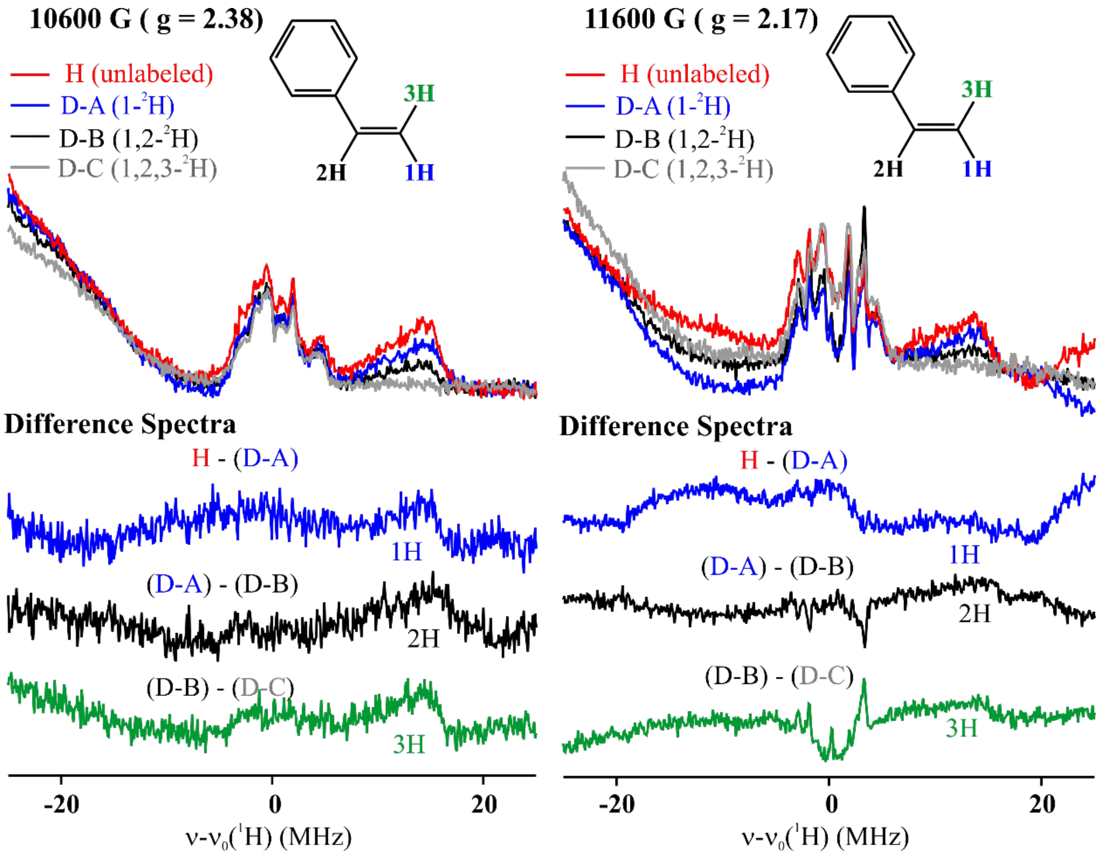

Figure S1. Q-band CW stochastic ^1H ENDOR spectra of unlabeled and ^2H labeled styrene at higher magnetic fields, as indicated. Conditions are same as Figure 3. Difference ENDOR spectra, as indicated.

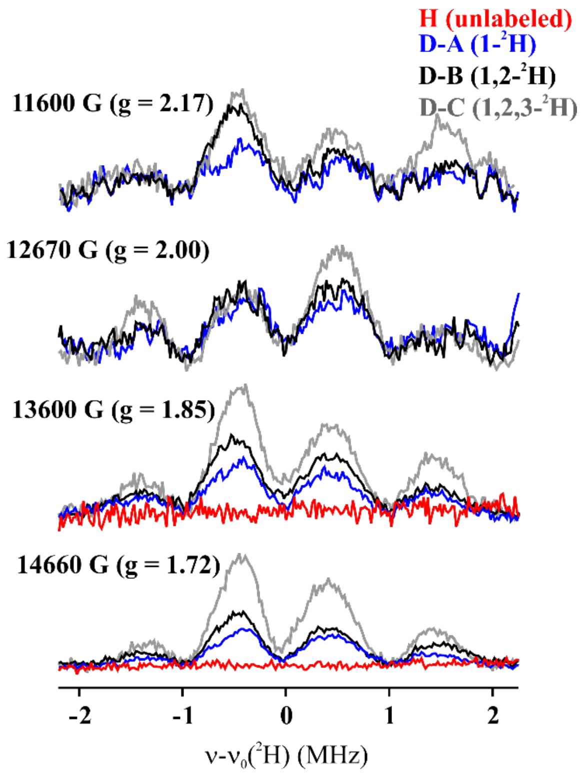

Figure S2. Q-band ^{2}H Mims ENDOR spectra of unlabeled and ^{2}H labeled styrene at higher fields, as indicated. *Conditions:* T = 2 K, microwave frequency = 34.95 GHz, MW pulse length (π/2) = 50 ns, τ = 500 ns.

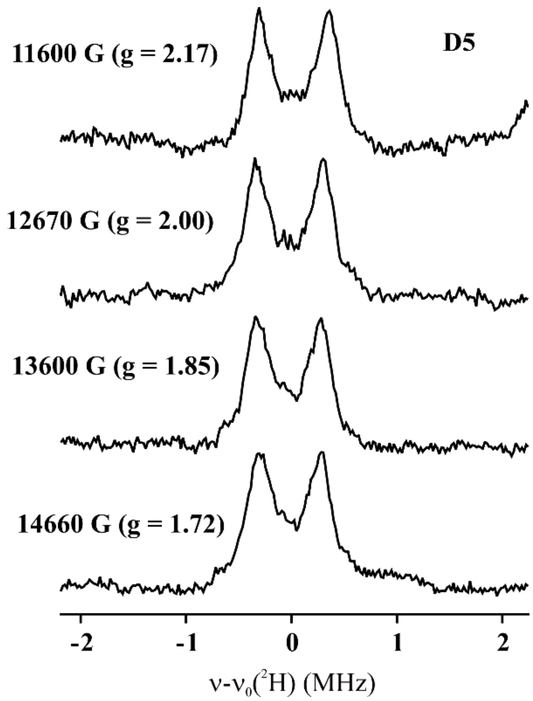

Figure S3. Q-band ^2H Mims ENDOR spectra of ^2H labeled in phenyl ring of styrene at higher fields. Conditions are same as Figure S2.

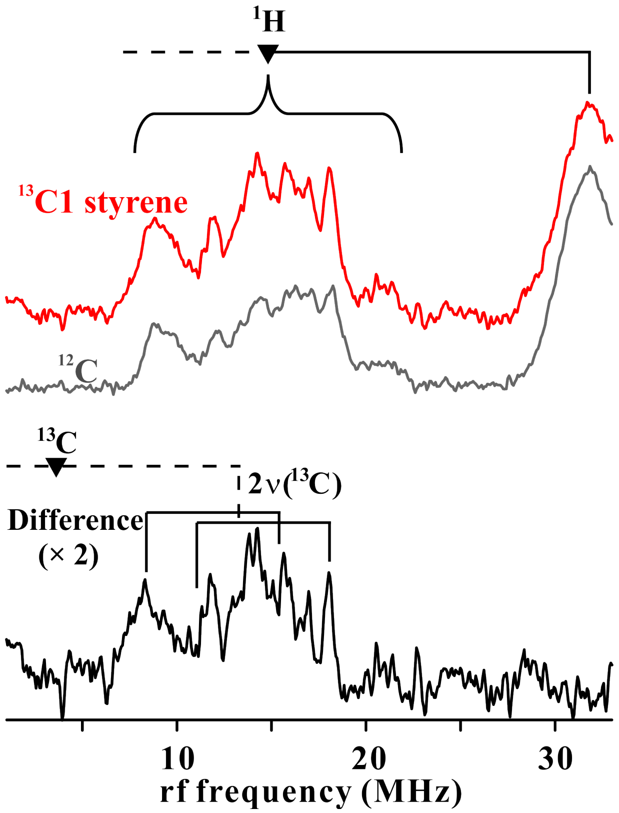

Figure S4. Q-band CW ^{13}C ENDOR spectra of **1** with ^{13}C1 labeled (red) and unlabeled (gray) styrene at g_1'. Their difference spectrum was shown in black and its intensity was multiplied by two. Larmor frequency of ^{13}C, ^{14}N and ^{1}H, 3.7, 1.1 and 14.8 MHz, respectively. *Conditions*: T = 2 K, microwave frequency = ~ 35.0 GHz, microwave power = 1 mW, 100 kHz modulation amplitude = 0.3 G, and rf sweep rate = 2 MHz/s.

Figure S5. ^{1}H NMR spectra of deuterated styrenes in $CDCl_3$.

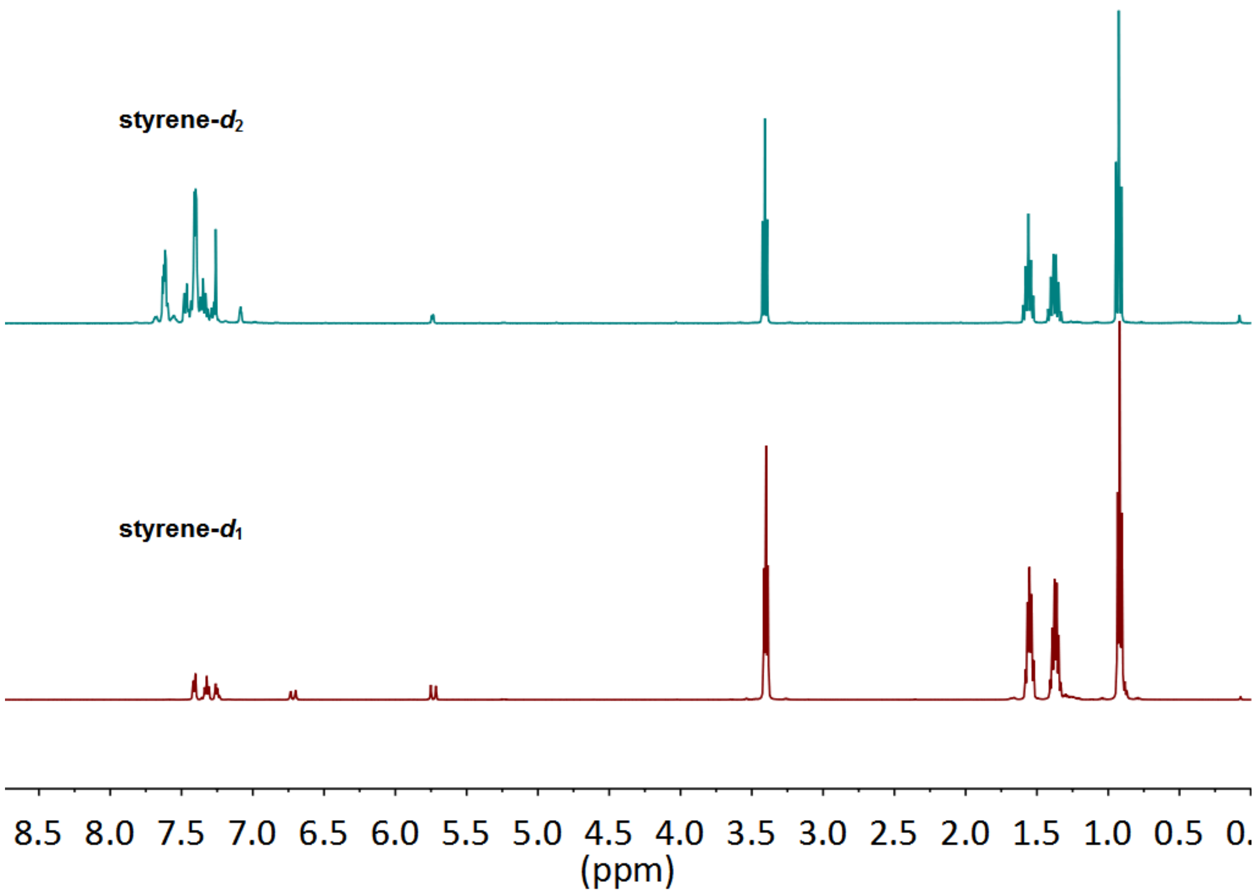

Figure S6. ^{1}H NMR spectra of deuterated styrene complexes in C_6D_{12}.

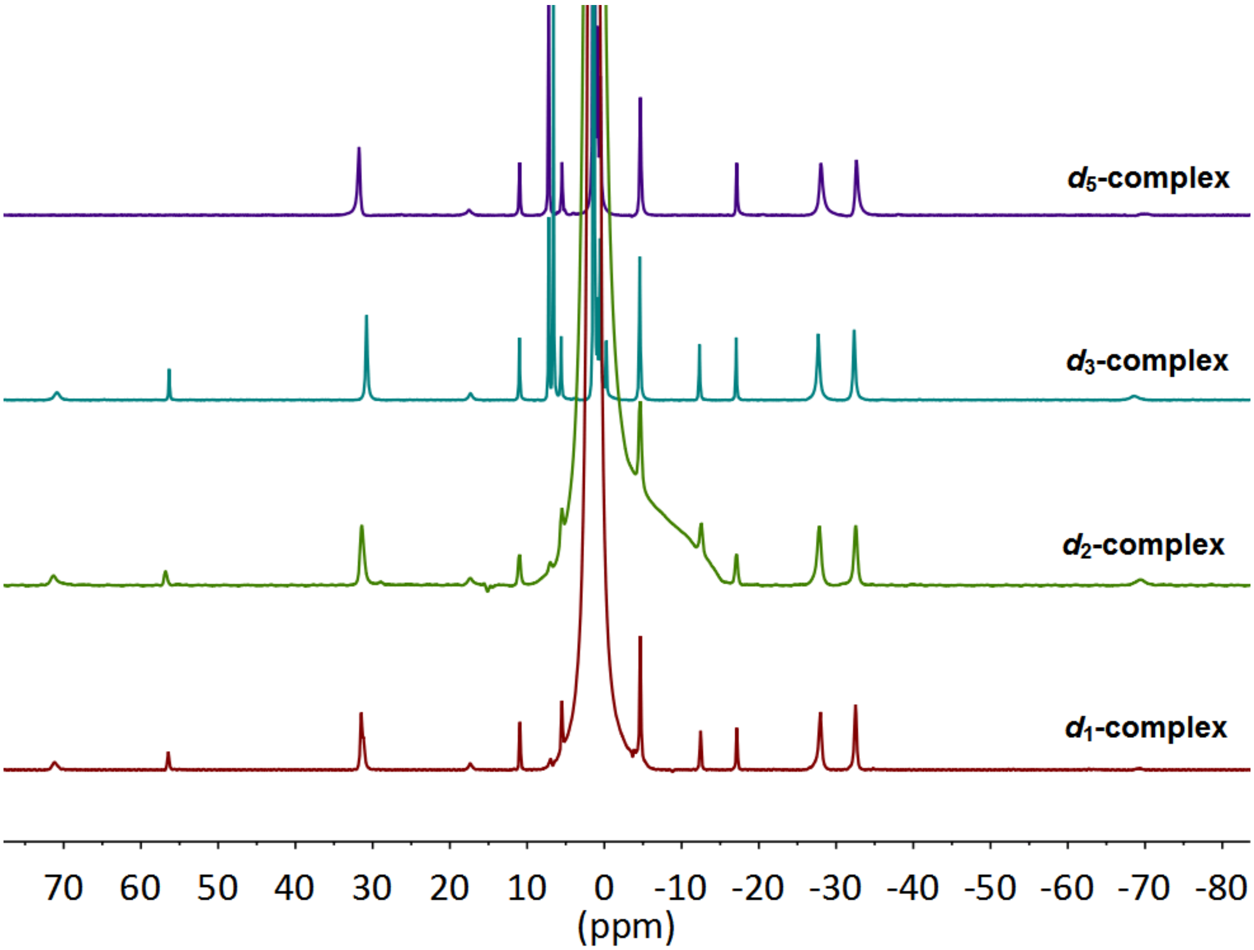

Table S1. Crystal data and structure refinement for **1**.

Identification code	mini-15066	
Empirical formula	$C_{80}H_{112}Fe_2N_4$	
Formula weight	1241.43	
Temperature	93(2) K	
Wavelength	0.71073 Å	
Crystal system	Monoclinic	
Space group	P 2_1/c	
Unit cell dimensions	a = 15.3867(11) Å	α = 90°.
	b = 20.3731(14) Å	β = 90.744(4)°.
	c = 22.3197(16) Å	γ = 90°.
Volume	6996.1(9) $Å^3$	
Z	4	
Density (calculated)	1.179 g/cm^3	
Absorption coefficient	0.460 mm^{-1}	
F(000)	2688	
Crystal size	0.200 x 0.100 x 0.050 mm^3	
Crystal color and habit	Yellow Plate	
Diffractometer	Rigaku Mercury275R CCD	
Theta range for data collection	1.659 to 24.712°.	
Index ranges	-18<=h<=18, -23<=k<=23, -26<=l<=26	
Reflections collected	95170	
Independent reflections	11912 [R(int) = 0.2190]	
Observed reflections (I > 2sigma(I))	6732	
Completeness to theta = 24.712°	99.9 %	
Absorption correction	Semi-empirical from equivalents	
Max. and min. transmission	0.977 and 0.605	
Solution method	SHELXT-2014/5 (Sheldrick, 2014)	
Refinement method	SHELXL-2014/7 (Sheldrick, 2014)	
Data / restraints / parameters	11912 / 40 / 836	

Goodness-of-fit on F^2	1.005
Final R indices [I>2sigma(I)]	R1 = 0.0655, wR2 = 0.1520
R indices (all data)	R1 = 0.1351, wR2 = 0.1946
Largest diff. peak and hole	0.854 and -0.668 e.$Å^{-3}$

Table S2. Atomic coordinates (x 10^4) and equivalent isotropic displacement parameters (Å^{2}x 10^3) for **1**. U(eq) is defined as one third of the trace of the orthogonalized U^{ij} tensor.

	x	y	z	U(eq)
Fe(1)	8497(1)	7750(1)	3102(1)	29(1)
N(11)	7614(2)	8373(2)	3366(2)	27(1)
N(21)	7978(2)	7592(2)	2300(2)	28(1)
C(11)	6176(3)	8885(2)	3344(2)	33(1)
C(21)	6871(3)	8461(2)	3073(2)	28(1)
C(31)	6680(3)	8180(2)	2520(2)	29(1)
C(41)	7198(3)	7813(2)	2137(2)	25(1)
C(51)	6865(3)	7711(2)	1515(2)	34(1)
C(12)	7815(3)	8767(2)	3875(2)	29(1)
C(22)	7572(3)	8571(2)	4452(2)	32(1)
C(32)	7833(3)	8956(2)	4932(2)	42(1)
C(42)	8312(3)	9517(2)	4846(2)	44(1)
C(52)	8539(3)	9701(2)	4279(2)	40(1)
C(62)	8320(3)	9332(2)	3788(2)	32(1)
C(72)	7043(3)	7961(2)	4562(2)	35(1)
C(82)	6225(3)	8113(2)	4915(2)	44(1)
C(92)	7576(4)	7443(2)	4886(2)	48(1)
C(102)	8598(3)	9530(2)	3164(2)	39(1)
C(112)	7934(4)	9957(3)	2858(3)	64(2)
C(122)	9472(3)	9864(3)	3150(3)	51(1)
C(13)	8484(3)	7261(2)	1857(2)	27(1)
C(23)	8489(3)	6570(2)	1842(2)	33(1)
C(33)	8999(3)	6260(2)	1424(2)	42(1)
C(43)	9493(3)	6613(3)	1025(2)	44(1)
C(53)	9490(3)	7287(3)	1046(2)	41(1)

C(63)	8986(3)	7620(2)	1459(2)	32(1)
C(73)	7952(3)	6178(2)	2268(2)	40(1)
C(83)	7058(4)	6031(3)	2005(3)	58(2)
C(93)	8386(4)	5543(2)	2468(3)	59(2)
C(103)	9009(3)	8363(2)	1485(2)	38(1)
C(113)	8924(4)	8678(3)	869(2)	54(2)
C(123)	9850(4)	8591(3)	1787(3)	57(2)
C(14)	9476(4)	7109(3)	3155(3)	58(2)
C(24)	9502(4)	7513(3)	3652(3)	49(1)
C(34)	10132(3)	8059(2)	3757(2)	40(1)
C(44)	10031(4)	8459(2)	4249(2)	42(1)
C(54)	10616(3)	8940(2)	4382(2)	43(1)
C(64)	11329(3)	9037(2)	4032(2)	40(1)
C(74)	11446(4)	8649(3)	3538(2)	45(1)
C(84)	10847(4)	8159(2)	3399(2)	45(1)
Fe(2)	3507(1)	7119(1)	3172(1)	28(1)
N(15)	2955(2)	7179(2)	2368(2)	27(1)
N(25)	2619(2)	6549(2)	3521(2)	26(1)
C(15)	1794(3)	6986(2)	1626(2)	36(1)
C(25)	2174(3)	6942(2)	2245(2)	27(1)
C(35)	1672(3)	6619(2)	2666(2)	29(1)
C(45)	1880(3)	6410(2)	3243(2)	27(1)
C(55)	1215(3)	5997(2)	3564(2)	37(1)
C(16)	3483(3)	7413(2)	1889(2)	28(1)
C(26)	3605(3)	8093(2)	1817(2)	31(1)
C(36)	4184(3)	8297(2)	1387(2)	37(1)
C(46)	4614(3)	7856(2)	1029(2)	39(1)
C(56)	4484(3)	7200(2)	1106(2)	35(1)
C(66)	3923(3)	6964(2)	1533(2)	32(1)
C(76)	3107(3)	8579(2)	2188(2)	34(1)

C(86)	2223(4)	8714(2)	1916(2)	47(1)
C(96)	3586(4)	9226(2)	2286(3)	52(2)
C(106)	3821(3)	6228(2)	1610(2)	37(1)
C(116)	3548(4)	5893(2)	1028(2)	50(1)
C(126)	4658(4)	5934(3)	1858(3)	56(2)
C(17)	2812(3)	6259(2)	4092(2)	29(1)
C(27)	2623(3)	6593(2)	4621(2)	31(1)
C(37)	2889(3)	6326(2)	5158(2)	34(1)
C(47)	3333(3)	5742(2)	5187(2)	38(1)
C(57)	3499(3)	5415(2)	4665(2)	37(1)
C(67)	3257(3)	5653(2)	4109(2)	32(1)
C(77)	2111(3)	7229(2)	4624(2)	34(1)
C(87)	2648(4)	7784(2)	4896(2)	46(1)
C(97)	1279(3)	7150(3)	4971(2)	45(1)
C(107)	3450(3)	5262(2)	3554(2)	40(1)
C(117)	4407(4)	5203(3)	3444(3)	56(2)
C(127)	3055(5)	4578(3)	3597(3)	70(2)
C(18)	4524(4)	7736(3)	3103(3)	54(2)
C(28)	4551(4)	7444(2)	3654(3)	48(1)
C(38)	5167(3)	6920(2)	3839(2)	38(1)
C(48)	5883(4)	6741(2)	3503(2)	45(1)
C(58)	6465(4)	6274(2)	3721(3)	46(1)
C(68)	6334(3)	5990(2)	4266(2)	45(1)
C(78)	5622(3)	6162(2)	4594(2)	44(1)
C(88)	5047(3)	6622(2)	4382(2)	39(1)
C(10)	8598(8)	5214(5)	4073(5)	98(4)
C(20)	8938(9)	4903(6)	4606(6)	94(4)
C(30)	9818(8)	5116(5)	4722(5)	93(4)
C(40)	8684(15)	5849(11)	4246(11)	90(8)
C(50)	8870(40)	5040(20)	4410(30)	140(30)

C(60)	9780(30)	5309(16)	4790(17)	93(4)
C(70)	4856(5)	9774(3)	4751(3)	71(2)
C(80)	4020(5)	9917(3)	4456(3)	78(2)
C(90)	3770(5)	9451(3)	3964(4)	86(2)

Table S3. Bond lengths [Å] and angles [°] for **1**.

Fe(1)-N(11)	1.955(4)
Fe(1)-N(21)	1.977(4)
Fe(1)-C(14)	1.996(6)
Fe(1)-C(24)	2.020(5)
N(11)-C(21)	1.322(6)
N(11)-C(12)	1.422(6)
N(21)-C(41)	1.329(6)
N(21)-C(13)	1.436(5)
C(11)-C(21)	1.508(6)
C(11)-H(11A)	0.9800
C(11)-H(11B)	0.9800
C(11)-H(11C)	0.9800
C(21)-C(31)	1.387(6)
C(31)-C(41)	1.393(6)
C(31)-H(31)	0.9500
C(41)-C(51)	1.489(6)
C(51)-H(51A)	0.9800
C(51)-H(51B)	0.9800
C(51)-H(51C)	0.9800
C(12)-C(22)	1.403(6)
C(12)-C(62)	1.404(6)
C(22)-C(32)	1.382(6)
C(22)-C(72)	1.508(6)
C(32)-C(42)	1.374(7)
C(32)-H(32)	0.9500
C(42)-C(52)	1.371(7)
C(42)-H(42)	0.9500
C(52)-C(62)	1.367(6)

C(52)-H(52)	0.9500
C(62)-C(102)	1.518(7)
C(72)-C(92)	1.515(7)
C(72)-C(82)	1.526(7)
C(72)-H(72)	1.0000
C(82)-H(82A)	0.9800
C(82)-H(82B)	0.9800
C(82)-H(82C)	0.9800
C(92)-H(92A)	0.9800
C(92)-H(92B)	0.9800
C(92)-H(92C)	0.9800
C(102)-C(112)	1.499(7)
C(102)-C(122)	1.508(7)
C(102)-H(102)	1.0000
C(112)-H(11D)	0.9800
C(112)-H(11E)	0.9800
C(112)-H(11F)	0.9800
C(122)-H(12A)	0.9800
C(122)-H(12B)	0.9800
C(122)-H(12C)	0.9800
C(13)-C(63)	1.392(6)
C(13)-C(23)	1.409(6)
C(23)-C(33)	1.380(7)
C(23)-C(73)	1.498(7)
C(33)-C(43)	1.380(7)
C(33)-H(33)	0.9500
C(43)-C(53)	1.375(7)
C(43)-H(43)	0.9500
C(53)-C(63)	1.389(7)
C(53)-H(53)	0.9500

C(63)-C(103)	1.514(6)
C(73)-C(83)	1.518(7)
C(73)-C(93)	1.520(7)
C(73)-H(73)	1.0000
C(83)-H(83A)	0.9800
C(83)-H(83B)	0.9800
C(83)-H(83C)	0.9800
C(93)-H(93A)	0.9800
C(93)-H(93B)	0.9800
C(93)-H(93C)	0.9800
C(103)-C(113)	1.522(7)
C(103)-C(123)	1.523(7)
C(103)-H(103)	1.0000
C(113)-H(11G)	0.9800
C(113)-H(11H)	0.9800
C(113)-H(11I)	0.9800
C(123)-H(12D)	0.9800
C(123)-H(12E)	0.9800
C(123)-H(12F)	0.9800
C(14)-C(24)	1.381(8)
C(14)-H(14A)	1.08(6)
C(14)-H(14B)	1.01(9)
C(24)-C(34)	1.493(7)
C(24)-H(24)	0.9500
C(34)-C(44)	1.378(7)
C(34)-C(84)	1.382(8)
C(44)-C(54)	1.361(7)
C(44)-H(44)	0.9500
C(54)-C(64)	1.368(7)
C(54)-H(54)	0.9500

C(64)-C(74)	1.372(7)
C(64)-H(64)	0.9500
C(74)-C(84)	1.392(7)
C(74)-H(74)	0.9500
C(84)-H(84)	0.9500
Fe(2)-N(25)	1.963(4)
Fe(2)-N(15)	1.978(4)
Fe(2)-C(18)	2.014(5)
Fe(2)-C(28)	2.033(5)
N(15)-C(25)	1.320(6)
N(15)-C(16)	1.433(6)
N(25)-C(45)	1.319(5)
N(25)-C(17)	1.431(5)
C(15)-C(25)	1.497(6)
C(15)-H(15A)	0.9800
C(15)-H(15B)	0.9800
C(15)-H(15C)	0.9800
C(25)-C(35)	1.390(6)
C(35)-C(45)	1.389(6)
C(35)-H(35)	0.9500
C(45)-C(55)	1.511(6)
C(55)-H(55A)	0.9800
C(55)-H(55B)	0.9800
C(55)-H(55C)	0.9800
C(16)-C(66)	1.393(6)
C(16)-C(26)	1.406(6)
C(26)-C(36)	1.382(7)
C(26)-C(76)	1.508(6)
C(36)-C(46)	1.379(7)
C(36)-H(36)	0.9500

C(46)-C(56)	1.362(6)
C(46)-H(46)	0.9500
C(56)-C(66)	1.380(6)
C(56)-H(56)	0.9500
C(66)-C(106)	1.518(6)
C(76)-C(86)	1.507(7)
C(76)-C(96)	1.524(7)
C(76)-H(76)	1.0000
C(86)-H(86A)	0.9800
C(86)-H(86B)	0.9800
C(86)-H(86C)	0.9800
C(96)-H(96A)	0.9800
C(96)-H(96B)	0.9800
C(96)-H(96C)	0.9800
C(106)-C(126)	1.518(7)
C(106)-C(116)	1.523(7)
C(106)-H(106)	1.0000
C(116)-H(11J)	0.9800
C(116)-H(11K)	0.9800
C(116)-H(11L)	0.9800
C(126)-H(12G)	0.9800
C(126)-H(12H)	0.9800
C(126)-H(12I)	0.9800
C(17)-C(27)	1.398(6)
C(17)-C(67)	1.411(6)
C(27)-C(37)	1.373(6)
C(27)-C(77)	1.517(6)
C(37)-C(47)	1.374(6)
C(37)-H(37)	0.9500
C(47)-C(57)	1.370(7)

C(47)-H(47)	0.9500
C(57)-C(67)	1.379(6)
C(57)-H(57)	0.9500
C(67)-C(107)	1.506(6)
C(77)-C(97)	1.514(7)
C(77)-C(87)	1.521(7)
C(77)-H(77)	1.0000
C(87)-H(87A)	0.9800
C(87)-H(87B)	0.9800
C(87)-H(87C)	0.9800
C(97)-H(97A)	0.9800
C(97)-H(97B)	0.9800
C(97)-H(97C)	0.9800
C(107)-C(117)	1.501(7)
C(107)-C(127)	1.524(7)
C(107)-H(107)	1.0000
C(117)-H(11M)	0.9800
C(117)-H(11N)	0.9800
C(117)-H(11O)	0.9800
C(127)-H(12J)	0.9800
C(127)-H(12K)	0.9800
C(127)-H(12L)	0.9800
C(18)-C(28)	1.366(8)
C(18)-H(18A)	1.11(9)
C(18)-H(18B)	1.12(6)
C(28)-C(38)	1.482(7)
C(28)-H(28)	0.9500
C(38)-C(88)	1.370(7)
C(38)-C(48)	1.389(7)
C(48)-C(58)	1.391(7)

C(48)-H(48)	0.9500
C(58)-C(68)	1.364(7)
C(58)-H(58)	0.9500
C(68)-C(78)	1.371(7)
C(68)-H(68)	0.9500
C(78)-C(88)	1.370(7)
C(78)-H(78)	0.9500
C(88)-H(88)	0.9500
C(10)-C(20)	1.441(13)
C(10)-H(10A)	0.9800
C(10)-H(10B)	0.9800
C(10)-H(10C)	0.9800
C(20)-C(30)	1.442(13)
C(20)-H(20A)	0.9900
C(20)-H(20B)	0.9900
C(30)-C(30)#1	1.437(16)
C(30)-H(30A)	0.9900
C(30)-H(30B)	0.9900
C(40)-C(50)	1.71(4)
C(40)-H(40A)	0.9800
C(40)-H(40B)	0.9800
C(40)-H(40C)	0.9800
C(50)-C(60)	1.71(4)
C(50)-H(50A)	0.9900
C(50)-H(50B)	0.9900
C(60)-C(60)#1	1.71(4)
C(60)-H(60A)	0.9900
C(60)-H(60B)	0.9900
C(70)-C(80)	1.466(9)
C(70)-C(70)#2	1.505(13)

C(70)-H(70A)	0.9900
C(70)-H(70B)	0.9900
C(80)-C(90)	1.497(10)
C(80)-H(80A)	0.9900
C(80)-H(80B)	0.9900
C(90)-H(90A)	0.9800
C(90)-H(90B)	0.9800
C(90)-H(90C)	0.9800

N(11)-Fe(1)-N(21)	95.97(15)
N(11)-Fe(1)-C(14)	159.0(2)
N(21)-Fe(1)-C(14)	104.0(2)
N(11)-Fe(1)-C(24)	120.1(2)
N(21)-Fe(1)-C(24)	143.9(2)
C(14)-Fe(1)-C(24)	40.2(2)
C(21)-N(11)-C(12)	119.8(4)
C(21)-N(11)-Fe(1)	122.6(3)
C(12)-N(11)-Fe(1)	117.5(3)
C(41)-N(21)-C(13)	117.8(4)
C(41)-N(21)-Fe(1)	123.0(3)
C(13)-N(21)-Fe(1)	119.0(3)
C(21)-C(11)-H(11A)	109.5
C(21)-C(11)-H(11B)	109.5
H(11A)-C(11)-H(11B)	109.5
C(21)-C(11)-H(11C)	109.5
H(11A)-C(11)-H(11C)	109.5
H(11B)-C(11)-H(11C)	109.5
N(11)-C(21)-C(31)	123.8(4)
N(11)-C(21)-C(11)	119.4(4)
C(31)-C(21)-C(11)	116.8(4)

C(21)-C(31)-C(41)	130.5(4)
C(21)-C(31)-H(31)	114.7
C(41)-C(31)-H(31)	114.7
N(21)-C(41)-C(31)	122.4(4)
N(21)-C(41)-C(51)	120.4(4)
C(31)-C(41)-C(51)	117.1(4)
C(41)-C(51)-H(51A)	109.5
C(41)-C(51)-H(51B)	109.5
H(51A)-C(51)-H(51B)	109.5
C(41)-C(51)-H(51C)	109.5
H(51A)-C(51)-H(51C)	109.5
H(51B)-C(51)-H(51C)	109.5
C(22)-C(12)-C(62)	120.9(4)
C(22)-C(12)-N(11)	121.0(4)
C(62)-C(12)-N(11)	117.9(4)
C(32)-C(22)-C(12)	118.2(4)
C(32)-C(22)-C(72)	119.5(4)
C(12)-C(22)-C(72)	122.3(4)
C(42)-C(32)-C(22)	121.0(5)
C(42)-C(32)-H(32)	119.5
C(22)-C(32)-H(32)	119.5
C(52)-C(42)-C(32)	120.0(5)
C(52)-C(42)-H(42)	120.0
C(32)-C(42)-H(42)	120.0
C(62)-C(52)-C(42)	121.8(5)
C(62)-C(52)-H(52)	119.1
C(42)-C(52)-H(52)	119.1
C(52)-C(62)-C(12)	118.2(5)
C(52)-C(62)-C(102)	121.3(4)
C(12)-C(62)-C(102)	120.5(4)

C(22)-C(72)-C(92)	111.3(4)
C(22)-C(72)-C(82)	111.6(4)
C(92)-C(72)-C(82)	109.9(4)
C(22)-C(72)-H(72)	108.0
C(92)-C(72)-H(72)	108.0
C(82)-C(72)-H(72)	108.0
C(72)-C(82)-H(82A)	109.5
C(72)-C(82)-H(82B)	109.5
H(82A)-C(82)-H(82B)	109.5
C(72)-C(82)-H(82C)	109.5
H(82A)-C(82)-H(82C)	109.5
H(82B)-C(82)-H(82C)	109.5
C(72)-C(92)-H(92A)	109.5
C(72)-C(92)-H(92B)	109.5
H(92A)-C(92)-H(92B)	109.5
C(72)-C(92)-H(92C)	109.5
H(92A)-C(92)-H(92C)	109.5
H(92B)-C(92)-H(92C)	109.5
C(112)-C(102)-C(122)	109.3(4)
C(112)-C(102)-C(62)	111.9(4)
C(122)-C(102)-C(62)	113.7(4)
C(112)-C(102)-H(102)	107.2
C(122)-C(102)-H(102)	107.2
C(62)-C(102)-H(102)	107.2
C(102)-C(112)-H(11D)	109.5
C(102)-C(112)-H(11E)	109.5
H(11D)-C(112)-H(11E)	109.5
C(102)-C(112)-H(11F)	109.5
H(11D)-C(112)-H(11F)	109.5
H(11E)-C(112)-H(11F)	109.5

C(102)-C(122)-H(12A)	109.5
C(102)-C(122)-H(12B)	109.5
H(12A)-C(122)-H(12B)	109.5
C(102)-C(122)-H(12C)	109.5
H(12A)-C(122)-H(12C)	109.5
H(12B)-C(122)-H(12C)	109.5
C(63)-C(13)-C(23)	120.5(4)
C(63)-C(13)-N(21)	120.2(4)
C(23)-C(13)-N(21)	119.2(4)
C(33)-C(23)-C(13)	118.5(4)
C(33)-C(23)-C(73)	120.5(4)
C(13)-C(23)-C(73)	121.0(4)
C(23)-C(33)-C(43)	121.4(5)
C(23)-C(33)-H(33)	119.3
C(43)-C(33)-H(33)	119.3
C(53)-C(43)-C(33)	119.8(5)
C(53)-C(43)-H(43)	120.1
C(33)-C(43)-H(43)	120.1
C(43)-C(53)-C(63)	120.9(5)
C(43)-C(53)-H(53)	119.6
C(63)-C(53)-H(53)	119.6
C(53)-C(63)-C(13)	119.1(4)
C(53)-C(63)-C(103)	120.0(4)
C(13)-C(63)-C(103)	120.9(4)
C(23)-C(73)-C(83)	111.4(4)
C(23)-C(73)-C(93)	113.4(4)
C(83)-C(73)-C(93)	109.7(4)
C(23)-C(73)-H(73)	107.4
C(83)-C(73)-H(73)	107.4
C(93)-C(73)-H(73)	107.4

C(73)-C(83)-H(83A)	109.5
C(73)-C(83)-H(83B)	109.5
H(83A)-C(83)-H(83B)	109.5
C(73)-C(83)-H(83C)	109.5
H(83A)-C(83)-H(83C)	109.5
H(83B)-C(83)-H(83C)	109.5
C(73)-C(93)-H(93A)	109.5
C(73)-C(93)-H(93B)	109.5
H(93A)-C(93)-H(93B)	109.5
C(73)-C(93)-H(93C)	109.5
H(93A)-C(93)-H(93C)	109.5
H(93B)-C(93)-H(93C)	109.5
C(63)-C(103)-C(113)	112.7(4)
C(63)-C(103)-C(123)	110.0(4)
C(113)-C(103)-C(123)	109.4(4)
C(63)-C(103)-H(103)	108.2
C(113)-C(103)-H(103)	108.2
C(123)-C(103)-H(103)	108.2
C(103)-C(113)-H(11G)	109.5
C(103)-C(113)-H(11H)	109.5
H(11G)-C(113)-H(11H)	109.5
C(103)-C(113)-H(11I)	109.5
H(11G)-C(113)-H(11I)	109.5
H(11H)-C(113)-H(11I)	109.5
C(103)-C(123)-H(12D)	109.5
C(103)-C(123)-H(12E)	109.5
H(12D)-C(123)-H(12E)	109.5
C(103)-C(123)-H(12F)	109.5
H(12D)-C(123)-H(12F)	109.5
H(12E)-C(123)-H(12F)	109.5

C(24)-C(14)-Fe(1)	70.8(3)
C(24)-C(14)-H(14A)	120(3)
Fe(1)-C(14)-H(14A)	109(3)
C(24)-C(14)-H(14B)	102(5)
Fe(1)-C(14)-H(14B)	103(5)
H(14A)-C(14)-H(14B)	134(6)
C(14)-C(24)-C(34)	125.6(6)
C(14)-C(24)-Fe(1)	68.9(3)
C(34)-C(24)-Fe(1)	114.1(3)
C(14)-C(24)-H(24)	117.2
C(34)-C(24)-H(24)	117.2
Fe(1)-C(24)-H(24)	87.0
C(44)-C(34)-C(84)	118.2(5)
C(44)-C(34)-C(24)	119.0(5)
C(84)-C(34)-C(24)	122.6(5)
C(54)-C(44)-C(34)	121.2(5)
C(54)-C(44)-H(44)	119.4
C(34)-C(44)-H(44)	119.4
C(44)-C(54)-C(64)	120.9(5)
C(44)-C(54)-H(54)	119.6
C(64)-C(54)-H(54)	119.6
C(54)-C(64)-C(74)	119.3(5)
C(54)-C(64)-H(64)	120.3
C(74)-C(64)-H(64)	120.3
C(64)-C(74)-C(84)	119.9(5)
C(64)-C(74)-H(74)	120.0
C(84)-C(74)-H(74)	120.0
C(34)-C(84)-C(74)	120.4(5)
C(34)-C(84)-H(84)	119.8
C(74)-C(84)-H(84)	119.8

N(25)-Fe(2)-N(15)	95.99(15)
N(25)-Fe(2)-C(18)	160.9(2)
N(15)-Fe(2)-C(18)	102.5(2)
N(25)-Fe(2)-C(28)	122.10(19)
N(15)-Fe(2)-C(28)	141.90(19)
C(18)-Fe(2)-C(28)	39.5(2)
C(25)-N(15)-C(16)	119.4(4)
C(25)-N(15)-Fe(2)	123.0(3)
C(16)-N(15)-Fe(2)	117.1(3)
C(45)-N(25)-C(17)	119.9(4)
C(45)-N(25)-Fe(2)	122.7(3)
C(17)-N(25)-Fe(2)	117.4(3)
C(25)-C(15)-H(15A)	109.5
C(25)-C(15)-H(15B)	109.5
H(15A)-C(15)-H(15B)	109.5
C(25)-C(15)-H(15C)	109.5
H(15A)-C(15)-H(15C)	109.5
H(15B)-C(15)-H(15C)	109.5
N(15)-C(25)-C(35)	123.0(4)
N(15)-C(25)-C(15)	120.9(4)
C(35)-C(25)-C(15)	116.0(4)
C(45)-C(35)-C(25)	130.5(4)
C(45)-C(35)-H(35)	114.8
C(25)-C(35)-H(35)	114.8
N(25)-C(45)-C(35)	123.8(4)
N(25)-C(45)-C(55)	118.7(4)
C(35)-C(45)-C(55)	117.4(4)
C(45)-C(55)-H(55A)	109.5
C(45)-C(55)-H(55B)	109.5
H(55A)-C(55)-H(55B)	109.5

C(45)-C(55)-H(55C)	109.5
H(55A)-C(55)-H(55C)	109.5
H(55B)-C(55)-H(55C)	109.5
C(66)-C(16)-C(26)	120.9(4)
C(66)-C(16)-N(15)	119.4(4)
C(26)-C(16)-N(15)	119.4(4)
C(36)-C(26)-C(16)	117.7(4)
C(36)-C(26)-C(76)	121.3(4)
C(16)-C(26)-C(76)	121.0(4)
C(46)-C(36)-C(26)	121.6(4)
C(46)-C(36)-H(36)	119.2
C(26)-C(36)-H(36)	119.2
C(56)-C(46)-C(36)	119.6(5)
C(56)-C(46)-H(46)	120.2
C(36)-C(46)-H(46)	120.2
C(46)-C(56)-C(66)	121.6(5)
C(46)-C(56)-H(56)	119.2
C(66)-C(56)-H(56)	119.2
C(56)-C(66)-C(16)	118.6(4)
C(56)-C(66)-C(106)	119.2(4)
C(16)-C(66)-C(106)	122.2(4)
C(86)-C(76)-C(26)	111.2(4)
C(86)-C(76)-C(96)	109.5(4)
C(26)-C(76)-C(96)	113.5(4)
C(86)-C(76)-H(76)	107.5
C(26)-C(76)-H(76)	107.5
C(96)-C(76)-H(76)	107.5
C(76)-C(86)-H(86A)	109.5
C(76)-C(86)-H(86B)	109.5
H(86A)-C(86)-H(86B)	109.5

C(76)-C(86)-H(86C)	109.5
H(86A)-C(86)-H(86C)	109.5
H(86B)-C(86)-H(86C)	109.5
C(76)-C(96)-H(96A)	109.5
C(76)-C(96)-H(96B)	109.5
H(96A)-C(96)-H(96B)	109.5
C(76)-C(96)-H(96C)	109.5
H(96A)-C(96)-H(96C)	109.5
H(96B)-C(96)-H(96C)	109.5
C(126)-C(106)-C(66)	110.1(4)
C(126)-C(106)-C(116)	111.0(4)
C(66)-C(106)-C(116)	112.0(4)
C(126)-C(106)-H(106)	107.9
C(66)-C(106)-H(106)	107.9
C(116)-C(106)-H(106)	107.9
C(106)-C(116)-H(11J)	109.5
C(106)-C(116)-H(11K)	109.5
H(11J)-C(116)-H(11K)	109.5
C(106)-C(116)-H(11L)	109.5
H(11J)-C(116)-H(11L)	109.5
H(11K)-C(116)-H(11L)	109.5
C(106)-C(126)-H(12G)	109.5
C(106)-C(126)-H(12H)	109.5
H(12G)-C(126)-H(12H)	109.5
C(106)-C(126)-H(12I)	109.5
H(12G)-C(126)-H(12I)	109.5
H(12H)-C(126)-H(12I)	109.5
C(27)-C(17)-C(67)	120.6(4)
C(27)-C(17)-N(25)	120.5(4)
C(67)-C(17)-N(25)	118.7(4)

C(37)-C(27)-C(17)	118.8(4)
C(37)-C(27)-C(77)	119.0(4)
C(17)-C(27)-C(77)	122.2(4)
C(27)-C(37)-C(47)	121.8(5)
C(27)-C(37)-H(37)	119.1
C(47)-C(37)-H(37)	119.1
C(57)-C(47)-C(37)	118.6(5)
C(57)-C(47)-H(47)	120.7
C(37)-C(47)-H(47)	120.7
C(47)-C(57)-C(67)	122.9(4)
C(47)-C(57)-H(57)	118.5
C(67)-C(57)-H(57)	118.5
C(57)-C(67)-C(17)	117.2(4)
C(57)-C(67)-C(107)	120.0(4)
C(17)-C(67)-C(107)	122.7(4)
C(97)-C(77)-C(27)	110.7(4)
C(97)-C(77)-C(87)	109.4(4)
C(27)-C(77)-C(87)	110.9(4)
C(97)-C(77)-H(77)	108.6
C(27)-C(77)-H(77)	108.6
C(87)-C(77)-H(77)	108.6
C(77)-C(87)-H(87A)	109.5
C(77)-C(87)-H(87B)	109.5
H(87A)-C(87)-H(87B)	109.5
C(77)-C(87)-H(87C)	109.5
H(87A)-C(87)-H(87C)	109.5
H(87B)-C(87)-H(87C)	109.5
C(77)-C(97)-H(97A)	109.5
C(77)-C(97)-H(97B)	109.5
H(97A)-C(97)-H(97B)	109.5

C(77)-C(97)-H(97C)	109.5
H(97A)-C(97)-H(97C)	109.5
H(97B)-C(97)-H(97C)	109.5
C(117)-C(107)-C(67)	112.5(4)
C(117)-C(107)-C(127)	109.2(4)
C(67)-C(107)-C(127)	110.5(4)
C(117)-C(107)-H(107)	108.2
C(67)-C(107)-H(107)	108.2
C(127)-C(107)-H(107)	108.2
C(107)-C(117)-H(11M)	109.5
C(107)-C(117)-H(11N)	109.5
H(11M)-C(117)-H(11N)	109.5
C(107)-C(117)-H(11O)	109.5
H(11M)-C(117)-H(11O)	109.5
H(11N)-C(117)-H(11O)	109.5
C(107)-C(127)-H(12J)	109.5
C(107)-C(127)-H(12K)	109.5
H(12J)-C(127)-H(12K)	109.5
C(107)-C(127)-H(12L)	109.5
H(12J)-C(127)-H(12L)	109.5
H(12K)-C(127)-H(12L)	109.5
C(28)-C(18)-Fe(2)	71.0(3)
C(28)-C(18)-H(18A)	111(4)
Fe(2)-C(18)-H(18A)	106(4)
C(28)-C(18)-H(18B)	124(3)
Fe(2)-C(18)-H(18B)	112(3)
H(18A)-C(18)-H(18B)	120(5)
C(18)-C(28)-C(38)	125.2(6)
C(18)-C(28)-Fe(2)	69.5(3)
C(38)-C(28)-Fe(2)	114.3(3)

C(18)-C(28)-H(28)	117.4
C(38)-C(28)-H(28)	117.4
Fe(2)-C(28)-H(28)	86.2
C(88)-C(38)-C(48)	118.6(5)
C(88)-C(38)-C(28)	118.2(5)
C(48)-C(38)-C(28)	123.1(5)
C(38)-C(48)-C(58)	120.1(5)
C(38)-C(48)-H(48)	119.9
C(58)-C(48)-H(48)	119.9
C(68)-C(58)-C(48)	120.0(5)
C(68)-C(58)-H(58)	120.0
C(48)-C(58)-H(58)	120.0
C(58)-C(68)-C(78)	119.7(5)
C(58)-C(68)-H(68)	120.1
C(78)-C(68)-H(68)	120.1
C(88)-C(78)-C(68)	120.5(5)
C(88)-C(78)-H(78)	119.7
C(68)-C(78)-H(78)	119.7
C(38)-C(88)-C(78)	121.0(5)
C(38)-C(88)-H(88)	119.5
C(78)-C(88)-H(88)	119.5
C(20)-C(10)-H(10A)	109.5
C(20)-C(10)-H(10B)	109.5
H(10A)-C(10)-H(10B)	109.5
C(20)-C(10)-H(10C)	109.5
H(10A)-C(10)-H(10C)	109.5
H(10B)-C(10)-H(10C)	109.5
C(10)-C(20)-C(30)	110.1(12)
C(10)-C(20)-H(20A)	109.6
C(30)-C(20)-H(20A)	109.6

C(10)-C(20)-H(20B)	109.6
C(30)-C(20)-H(20B)	109.6
H(20A)-C(20)-H(20B)	108.1
C(30)#1-C(30)-C(20)	114.2(17)
C(30)#1-C(30)-H(30A)	108.7
C(20)-C(30)-H(30A)	108.7
C(30)#1-C(30)-H(30B)	108.7
C(20)-C(30)-H(30B)	108.7
H(30A)-C(30)-H(30B)	107.6
C(50)-C(40)-H(40A)	109.5
C(50)-C(40)-H(40B)	109.5
H(40A)-C(40)-H(40B)	109.5
C(50)-C(40)-H(40C)	109.5
H(40A)-C(40)-H(40C)	109.5
H(40B)-C(40)-H(40C)	109.5
C(40)-C(50)-C(60)	86(3)
C(40)-C(50)-H(50A)	114.3
C(60)-C(50)-H(50A)	114.3
C(40)-C(50)-H(50B)	114.3
C(60)-C(50)-H(50B)	114.3
H(50A)-C(50)-H(50B)	111.4
C(60)#1-C(60)-C(50)	111(4)
C(60)#1-C(60)-H(60A)	109.4
C(50)-C(60)-H(60A)	109.4
C(60)#1-C(60)-H(60B)	109.4
C(50)-C(60)-H(60B)	109.4
H(60A)-C(60)-H(60B)	108.0
C(80)-C(70)-C(70)#2	117.3(8)
C(80)-C(70)-H(70A)	108.0
C(70)#2-C(70)-H(70A)	108.0

C(80)-C(70)-H(70B)	108.0
C(70)#2-C(70)-H(70B)	108.0
H(70A)-C(70)-H(70B)	107.2
C(70)-C(80)-C(90)	114.8(6)
C(70)-C(80)-H(80A)	108.6
C(90)-C(80)-H(80A)	108.6
C(70)-C(80)-H(80B)	108.6
C(90)-C(80)-H(80B)	108.6
H(80A)-C(80)-H(80B)	107.5
C(80)-C(90)-H(90A)	109.5
C(80)-C(90)-H(90B)	109.5
H(90A)-C(90)-H(90B)	109.5
C(80)-C(90)-H(90C)	109.5
H(90A)-C(90)-H(90C)	109.5
H(90B)-C(90)-H(90C)	109.5

Symmetry transformations used to generate equivalent atoms:

#1 -x+2,-y+1,-z+1 #2 -x+1,-y+2,-z+1

Table S4. Anisotropic displacement parameters ($Å^2 x 10^3$) for **1**. The anisotropic displacement factor exponent takes the form: $-2p^2[h^2 a^{*2}U^{11} + ... + 2 h k a^* b^* U^{12}]$

	U^{11}	U^{22}	U^{33}	U^{23}	U^{13}	U^{12}
Fe(1)	30(1)	24(1)	32(1)	0(1)	1(1)	4(1)
N(11)	29(2)	19(2)	34(2)	0(2)	4(2)	-1(2)
N(21)	33(2)	22(2)	30(2)	1(2)	10(2)	1(2)
C(11)	29(3)	28(2)	41(3)	-5(2)	3(2)	3(2)
C(21)	29(3)	18(2)	37(3)	2(2)	6(2)	-6(2)
C(31)	28(3)	18(2)	41(3)	4(2)	-3(2)	-4(2)
C(41)	22(2)	20(2)	33(2)	4(2)	2(2)	-5(2)
C(51)	33(3)	34(2)	34(3)	-1(2)	4(2)	-1(2)
C(12)	28(3)	20(2)	39(3)	0(2)	6(2)	3(2)
C(22)	32(3)	26(2)	38(3)	-5(2)	6(2)	0(2)
C(32)	46(3)	40(3)	41(3)	-8(2)	11(3)	-4(2)
C(42)	42(3)	38(3)	53(4)	-22(3)	0(3)	-4(2)
C(52)	44(3)	23(2)	53(3)	-5(2)	4(3)	-1(2)
C(62)	31(3)	20(2)	46(3)	-1(2)	-1(2)	4(2)
C(72)	37(3)	33(3)	34(3)	0(2)	9(2)	-7(2)
C(82)	47(3)	41(3)	44(3)	-3(2)	7(3)	-8(3)
C(92)	55(4)	41(3)	49(3)	4(3)	12(3)	0(3)
C(102)	42(3)	22(2)	53(3)	4(2)	1(3)	-9(2)
C(112)	43(4)	67(4)	81(4)	37(3)	-4(3)	-8(3)
C(122)	43(3)	47(3)	64(4)	10(3)	6(3)	-4(3)
C(13)	20(2)	30(2)	32(2)	-5(2)	-1(2)	1(2)
C(23)	34(3)	30(2)	34(3)	-5(2)	0(2)	2(2)
C(33)	43(3)	36(3)	47(3)	-9(2)	-1(3)	8(2)
C(43)	38(3)	55(3)	39(3)	-10(3)	5(2)	13(3)
C(53)	29(3)	63(4)	32(3)	5(3)	3(2)	7(3)

C(63)	25(3)	36(3)	36(3)	2(2)	-1(2)	-1(2)
C(73)	42(3)	25(2)	52(3)	-6(2)	3(3)	-1(2)
C(83)	48(4)	43(3)	84(5)	8(3)	-3(3)	-11(3)
C(93)	67(4)	38(3)	73(4)	14(3)	16(3)	7(3)
C(103)	37(3)	36(3)	42(3)	5(2)	3(2)	-4(2)
C(113)	65(4)	42(3)	56(4)	12(3)	9(3)	-6(3)
C(123)	50(4)	52(3)	69(4)	5(3)	-1(3)	-9(3)
C(14)	50(4)	53(4)	70(5)	-5(3)	-14(3)	16(3)
C(24)	44(3)	47(3)	57(4)	-4(3)	-16(3)	10(3)
C(34)	33(3)	32(3)	54(3)	2(2)	-12(3)	8(2)
C(44)	45(3)	35(3)	47(3)	0(2)	-2(3)	4(2)
C(54)	38(3)	41(3)	49(3)	-5(2)	-2(3)	2(2)
C(64)	34(3)	35(3)	52(3)	-8(2)	1(3)	-4(2)
C(74)	44(3)	51(3)	39(3)	-3(3)	6(3)	6(3)
C(84)	58(4)	34(3)	42(3)	-10(2)	-11(3)	16(3)
Fe(2)	28(1)	25(1)	31(1)	1(1)	2(1)	-3(1)
N(15)	26(2)	23(2)	31(2)	-1(2)	5(2)	1(2)
N(25)	28(2)	21(2)	30(2)	-2(2)	4(2)	0(2)
C(15)	34(3)	34(3)	39(3)	-5(2)	1(2)	6(2)
C(25)	28(3)	24(2)	30(3)	-5(2)	5(2)	7(2)
C(35)	22(2)	31(2)	35(3)	-9(2)	2(2)	4(2)
C(45)	30(3)	21(2)	31(3)	-7(2)	6(2)	0(2)
C(55)	36(3)	35(3)	40(3)	1(2)	11(2)	-5(2)
C(16)	23(2)	33(2)	28(2)	2(2)	3(2)	1(2)
C(26)	31(3)	31(2)	31(3)	2(2)	1(2)	2(2)
C(36)	39(3)	34(3)	39(3)	6(2)	0(2)	-2(2)
C(46)	32(3)	53(3)	32(3)	8(2)	5(2)	2(2)
C(56)	33(3)	36(3)	37(3)	0(2)	3(2)	8(2)
C(66)	23(3)	40(3)	33(3)	6(2)	4(2)	5(2)
C(76)	41(3)	28(2)	35(3)	4(2)	2(2)	4(2)

C(86)	49(4)	42(3)	48(3)	1(2)	1(3)	12(3)
C(96)	54(4)	31(3)	70(4)	-4(3)	5(3)	-1(3)
C(106)	40(3)	31(2)	39(3)	-1(2)	6(2)	9(2)
C(116)	66(4)	36(3)	47(3)	-4(2)	10(3)	9(3)
C(126)	57(4)	47(3)	65(4)	3(3)	9(3)	7(3)
C(17)	33(3)	21(2)	33(3)	5(2)	1(2)	-7(2)
C(27)	30(3)	25(2)	38(3)	0(2)	7(2)	0(2)
C(37)	41(3)	31(2)	30(3)	2(2)	6(2)	-4(2)
C(47)	46(3)	31(2)	36(3)	7(2)	-2(2)	-2(2)
C(57)	43(3)	21(2)	48(3)	9(2)	-3(2)	-3(2)
C(67)	33(3)	23(2)	42(3)	-2(2)	5(2)	-4(2)
C(77)	42(3)	29(2)	30(3)	-1(2)	7(2)	10(2)
C(87)	58(4)	31(3)	48(3)	-5(2)	9(3)	9(3)
C(97)	44(3)	45(3)	47(3)	-3(3)	8(3)	9(3)
C(107)	53(4)	23(2)	42(3)	-6(2)	2(3)	0(2)
C(117)	67(4)	50(3)	51(4)	-19(3)	14(3)	0(3)
C(127)	95(5)	36(3)	78(5)	-16(3)	15(4)	-12(3)
C(18)	43(4)	51(4)	68(4)	16(3)	-14(3)	-20(3)
C(28)	45(3)	37(3)	60(4)	5(3)	-16(3)	-13(3)
C(38)	35(3)	29(2)	50(3)	0(2)	-10(3)	-6(2)
C(48)	56(4)	33(3)	47(3)	7(2)	-4(3)	-15(3)
C(58)	41(3)	38(3)	61(4)	-1(3)	12(3)	3(2)
C(68)	41(3)	31(3)	63(4)	13(3)	6(3)	4(2)
C(78)	45(3)	34(3)	54(3)	12(2)	1(3)	-1(2)
C(88)	30(3)	31(2)	56(3)	4(2)	-2(2)	0(2)
C(10)	111(8)	71(7)	113(8)	-40(6)	63(6)	-19(6)
C(20)	109(8)	57(6)	117(8)	-34(6)	74(6)	-40(6)
C(30)	103(6)	48(7)	129(8)	-64(6)	79(6)	-30(5)
C(40)	79(13)	80(15)	113(17)	-44(12)	73(11)	-40(10)
C(50)	150(20)	77(16)	210(60)	-40(15)	20(40)	-46(11)

C(60)	103(6)	48(7)	129(8)	-64(6)	79(6)	-30(5)
C(70)	83(5)	59(4)	70(5)	28(3)	35(4)	7(4)
C(80)	77(5)	71(5)	86(5)	27(4)	30(4)	21(4)
C(90)	87(6)	68(4)	103(6)	5(4)	15(5)	-5(4)

Table 5. Hydrogen coordinates (x 10^4) and isotropic displacement parameters ($Å^2$x 10^3) for **1**.

	x	y	z	U(eq)
H(11A)	5826	8621	3618	49
H(11B)	5801	9063	3025	49
H(11C)	6449	9247	3566	49
H(31)	6102	8250	2379	35
H(51A)	7080	8062	1256	51
H(51B)	6228	7717	1513	51
H(51C)	7068	7286	1366	51
H(32)	7678	8831	5326	51
H(42)	8485	9776	5181	53
H(52)	8857	10096	4225	48
H(72)	6860	7778	4165	42
H(82A)	6389	8291	5309	66
H(82B)	5888	7710	4968	66
H(82C)	5873	8436	4696	66
H(92A)	8059	7305	4633	72
H(92B)	7207	7063	4973	72
H(92C)	7807	7624	5262	72
H(102)	8646	9118	2922	47
H(11D)	7384	9717	2819	95
H(11E)	8139	10078	2459	95
H(11F)	7845	10355	3096	95
H(12A)	9439	10285	3361	77
H(12B)	9637	9941	2733	77
H(12C)	9908	9585	3347	77

H(33)	9009	5794	1410	51
H(43)	9833	6390	737	53
H(53)	9839	7529	776	49
H(73)	7867	6453	2633	48
H(83A)	6745	6443	1936	87
H(83B)	6733	5757	2286	87
H(83C)	7120	5796	1624	87
H(93A)	8421	5242	2126	89
H(93B)	8043	5340	2785	89
H(93C)	8973	5638	2620	89
H(103)	8511	8514	1733	46
H(11G)	9435	8568	631	81
H(11H)	8884	9156	913	81
H(11I)	8399	8514	666	81
H(12D)	9906	8386	2183	85
H(12E)	9838	9070	1832	85
H(12F)	10345	8465	1540	85
H(24)	9083	7437	3953	59
H(44)	9544	8398	4500	51
H(54)	10528	9212	4721	52
H(64)	11738	9371	4131	48
H(74)	11937	8714	3291	54
H(84)	10929	7891	3056	54
H(15A)	2087	7335	1403	54
H(15B)	1172	7085	1649	54
H(15C)	1873	6566	1419	54
H(35)	1094	6525	2539	35
H(55A)	1486	5588	3701	55
H(55B)	731	5896	3289	55
H(55C)	997	6240	3910	55

H(36)	4288	8754	1337	45
H(46)	4999	8008	730	47
H(56)	4787	6898	861	42
H(76)	3015	8377	2590	41
H(86A)	2290	8901	1514	70
H(86B)	1910	9026	2169	70
H(86C)	1893	8303	1888	70
H(96A)	4174	9137	2440	78
H(96B)	3271	9493	2577	78
H(96C)	3622	9463	1906	78
H(106)	3355	6151	1910	44
H(11J)	2977	6058	899	74
H(11K)	3515	5417	1091	74
H(11L)	3975	5987	718	74
H(12G)	5131	6014	1577	85
H(12H)	4583	5460	1913	85
H(12I)	4801	6138	2245	85
H(37)	2762	6552	5519	41
H(47)	3522	5569	5562	45
H(57)	3792	5005	4687	45
H(77)	1958	7347	4200	40
H(87A)	2769	7689	5319	68
H(87B)	2323	8197	4862	68
H(87C)	3197	7824	4681	68
H(97A)	931	6796	4793	68
H(97B)	948	7561	4955	68
H(97C)	1420	7043	5389	68
H(107)	3177	5492	3203	47
H(11M)	4653	5640	3376	84
H(11N)	4501	4928	3090	84

H(11O)	4692	5001	3793	84
H(12J)	3362	4326	3907	104
H(12K)	3108	4354	3210	104
H(12L)	2439	4614	3699	104
H(28)	4143	7588	3941	57
H(48)	5975	6939	3124	54
H(58)	6953	6152	3490	56
H(68)	6734	5674	4418	54
H(78)	5528	5961	4972	53
H(88)	4557	6737	4615	47
H(10A)	8584	5691	4132	147
H(10B)	8008	5054	3991	147
H(10C)	8971	5110	3734	147
H(20A)	8925	4421	4555	112
H(20B)	8570	5017	4952	112
H(30A)	10188	4964	4390	111
H(30B)	9831	5602	4722	111
H(40A)	8274	6029	4536	135
H(40B)	8437	5888	3841	135
H(40C)	9231	6093	4271	135
H(50A)	9002	4768	4050	173
H(50B)	8427	4835	4659	173
H(60A)	10211	5473	4504	111
H(60B)	9614	5677	5056	111
H(70A)	5311	9776	4441	85
H(70B)	4829	9323	4915	85
H(80A)	4043	10366	4288	93
H(80B)	3560	9911	4762	93
H(90A)	4219	9453	3657	129
H(90B)	3214	9589	3785	129

H(90C)	3711	9008	4129	129
H(14A)	9920(40)	7200(30)	2790(30)	68(19)
H(14B)	9190(60)	6700(40)	3320(40)	140(30)
H(18A)	4250(50)	8240(40)	3130(40)	130(30)
H(18B)	4960(40)	7600(30)	2730(30)	80(20)

Table S6. Torsion angles [°] for **1**.

C(12)-N(11)-C(21)-C(31)	169.0(4)
Fe(1)-N(11)-C(21)-C(31)	-7.5(6)
C(12)-N(11)-C(21)-C(11)	-11.2(6)
Fe(1)-N(11)-C(21)-C(11)	172.3(3)
N(11)-C(21)-C(31)-C(41)	-5.8(7)
C(11)-C(21)-C(31)-C(41)	174.4(4)
C(13)-N(21)-C(41)-C(31)	-175.4(4)
Fe(1)-N(21)-C(41)-C(31)	0.4(6)
C(13)-N(21)-C(41)-C(51)	0.6(6)
Fe(1)-N(21)-C(41)-C(51)	176.4(3)
C(21)-C(31)-C(41)-N(21)	9.8(7)
C(21)-C(31)-C(41)-C(51)	-166.4(4)
C(21)-N(11)-C(12)-C(22)	89.8(5)
Fe(1)-N(11)-C(12)-C(22)	-93.5(5)
C(21)-N(11)-C(12)-C(62)	-94.9(5)
Fe(1)-N(11)-C(12)-C(62)	81.7(4)
C(62)-C(12)-C(22)-C(32)	1.0(7)
N(11)-C(12)-C(22)-C(32)	176.1(4)
C(62)-C(12)-C(22)-C(72)	-178.9(4)
N(11)-C(12)-C(22)-C(72)	-3.9(7)
C(12)-C(22)-C(32)-C(42)	0.3(7)
C(72)-C(22)-C(32)-C(42)	-179.7(5)
C(22)-C(32)-C(42)-C(52)	-0.1(8)
C(32)-C(42)-C(52)-C(62)	-1.6(8)
C(42)-C(52)-C(62)-C(12)	2.9(7)
C(42)-C(52)-C(62)-C(102)	-178.1(5)
C(22)-C(12)-C(62)-C(52)	-2.6(7)
N(11)-C(12)-C(62)-C(52)	-177.9(4)

C(22)-C(12)-C(62)-C(102)	178.4(4)
N(11)-C(12)-C(62)-C(102)	3.1(6)
C(32)-C(22)-C(72)-C(92)	-68.1(6)
C(12)-C(22)-C(72)-C(92)	111.9(5)
C(32)-C(22)-C(72)-C(82)	55.1(6)
C(12)-C(22)-C(72)-C(82)	-125.0(5)
C(52)-C(62)-C(102)-C(112)	-90.8(6)
C(12)-C(62)-C(102)-C(112)	88.2(5)
C(52)-C(62)-C(102)-C(122)	33.6(6)
C(12)-C(62)-C(102)-C(122)	-147.4(4)
C(41)-N(21)-C(13)-C(63)	83.9(5)
Fe(1)-N(21)-C(13)-C(63)	-92.1(4)
C(41)-N(21)-C(13)-C(23)	-98.2(5)
Fe(1)-N(21)-C(13)-C(23)	85.8(4)
C(63)-C(13)-C(23)-C(33)	-0.3(7)
N(21)-C(13)-C(23)-C(33)	-178.2(4)
C(63)-C(13)-C(23)-C(73)	180.0(4)
N(21)-C(13)-C(23)-C(73)	2.1(7)
C(13)-C(23)-C(33)-C(43)	-0.2(7)
C(73)-C(23)-C(33)-C(43)	179.5(5)
C(23)-C(33)-C(43)-C(53)	0.8(8)
C(33)-C(43)-C(53)-C(63)	-1.0(8)
C(43)-C(53)-C(63)-C(13)	0.5(7)
C(43)-C(53)-C(63)-C(103)	178.5(5)
C(23)-C(13)-C(63)-C(53)	0.1(7)
N(21)-C(13)-C(63)-C(53)	178.0(4)
C(23)-C(13)-C(63)-C(103)	-177.9(4)
N(21)-C(13)-C(63)-C(103)	0.0(6)
C(33)-C(23)-C(73)-C(83)	-88.8(6)
C(13)-C(23)-C(73)-C(83)	91.0(5)

C(33)-C(23)-C(73)-C(93)	35.5(7)
C(13)-C(23)-C(73)-C(93)	-144.7(5)
C(53)-C(63)-C(103)-C(113)	47.1(6)
C(13)-C(63)-C(103)-C(113)	-134.9(5)
C(53)-C(63)-C(103)-C(123)	-75.2(6)
C(13)-C(63)-C(103)-C(123)	102.8(5)
Fe(1)-C(14)-C(24)-C(34)	-105.2(5)
C(14)-C(24)-C(34)-C(44)	174.2(5)
Fe(1)-C(24)-C(34)-C(44)	93.7(5)
C(14)-C(24)-C(34)-C(84)	-9.7(8)
Fe(1)-C(24)-C(34)-C(84)	-90.2(5)
C(84)-C(34)-C(44)-C(54)	0.0(7)
C(24)-C(34)-C(44)-C(54)	176.3(4)
C(34)-C(44)-C(54)-C(64)	-0.7(8)
C(44)-C(54)-C(64)-C(74)	0.9(8)
C(54)-C(64)-C(74)-C(84)	-0.5(8)
C(44)-C(34)-C(84)-C(74)	0.3(7)
C(24)-C(34)-C(84)-C(74)	-175.8(4)
C(64)-C(74)-C(84)-C(34)	-0.1(8)
C(16)-N(15)-C(25)-C(35)	170.3(4)
Fe(2)-N(15)-C(25)-C(35)	-1.2(6)
C(16)-N(15)-C(25)-C(15)	-7.1(6)
Fe(2)-N(15)-C(25)-C(15)	-178.5(3)
N(15)-C(25)-C(35)-C(45)	-8.2(7)
C(15)-C(25)-C(35)-C(45)	169.3(4)
C(17)-N(25)-C(45)-C(35)	-173.8(4)
Fe(2)-N(25)-C(45)-C(35)	3.8(6)
C(17)-N(25)-C(45)-C(55)	5.9(6)
Fe(2)-N(25)-C(45)-C(55)	-176.5(3)
C(25)-C(35)-C(45)-N(25)	6.8(7)

C(25)-C(35)-C(45)-C(55)	-172.9(4)
C(25)-N(15)-C(16)-C(66)	-78.5(5)
Fe(2)-N(15)-C(16)-C(66)	93.5(4)
C(25)-N(15)-C(16)-C(26)	106.6(5)
Fe(2)-N(15)-C(16)-C(26)	-81.4(4)
C(66)-C(16)-C(26)-C(36)	-0.9(7)
N(15)-C(16)-C(26)-C(36)	173.9(4)
C(66)-C(16)-C(26)-C(76)	177.9(4)
N(15)-C(16)-C(26)-C(76)	-7.3(7)
C(16)-C(26)-C(36)-C(46)	1.6(7)
C(76)-C(26)-C(36)-C(46)	-177.2(4)
C(26)-C(36)-C(46)-C(56)	-1.5(8)
C(36)-C(46)-C(56)-C(66)	0.6(7)
C(46)-C(56)-C(66)-C(16)	0.1(7)
C(46)-C(56)-C(66)-C(106)	-178.4(4)
C(26)-C(16)-C(66)-C(56)	0.1(7)
N(15)-C(16)-C(66)-C(56)	-174.7(4)
C(26)-C(16)-C(66)-C(106)	178.5(4)
N(15)-C(16)-C(66)-C(106)	3.7(7)
C(36)-C(26)-C(76)-C(86)	94.6(5)
C(16)-C(26)-C(76)-C(86)	-84.2(5)
C(36)-C(26)-C(76)-C(96)	-29.3(6)
C(16)-C(26)-C(76)-C(96)	151.9(5)
C(56)-C(66)-C(106)-C(126)	67.5(6)
C(16)-C(66)-C(106)-C(126)	-110.9(5)
C(56)-C(66)-C(106)-C(116)	-56.5(6)
C(16)-C(66)-C(106)-C(116)	125.1(5)
C(45)-N(25)-C(17)-C(27)	-94.7(5)
Fe(2)-N(25)-C(17)-C(27)	87.6(5)
C(45)-N(25)-C(17)-C(67)	90.1(5)

Fe(2)-N(25)-C(17)-C(67)	-87.6(4)
C(67)-C(17)-C(27)-C(37)	0.9(7)
N(25)-C(17)-C(27)-C(37)	-174.2(4)
C(67)-C(17)-C(27)-C(77)	-177.2(4)
N(25)-C(17)-C(27)-C(77)	7.7(7)
C(17)-C(27)-C(37)-C(47)	0.0(7)
C(77)-C(27)-C(37)-C(47)	178.2(4)
C(27)-C(37)-C(47)-C(57)	-1.2(7)
C(37)-C(47)-C(57)-C(67)	1.7(8)
C(47)-C(57)-C(67)-C(17)	-0.8(7)
C(47)-C(57)-C(67)-C(107)	-179.2(5)
C(27)-C(17)-C(67)-C(57)	-0.5(7)
N(25)-C(17)-C(67)-C(57)	174.6(4)
C(27)-C(17)-C(67)-C(107)	177.9(4)
N(25)-C(17)-C(67)-C(107)	-7.0(7)
C(37)-C(27)-C(77)-C(97)	-59.5(6)
C(17)-C(27)-C(77)-C(97)	118.7(5)
C(37)-C(27)-C(77)-C(87)	62.2(6)
C(17)-C(27)-C(77)-C(87)	-119.7(5)
C(57)-C(67)-C(107)-C(117)	-66.0(6)
C(17)-C(67)-C(107)-C(117)	115.7(5)
C(57)-C(67)-C(107)-C(127)	56.3(6)
C(17)-C(67)-C(107)-C(127)	-122.0(5)
Fe(2)-C(18)-C(28)-C(38)	105.9(5)
C(18)-C(28)-C(38)-C(88)	-172.4(5)
Fe(2)-C(28)-C(38)-C(88)	-91.1(5)
C(18)-C(28)-C(38)-C(48)	11.1(8)
Fe(2)-C(28)-C(38)-C(48)	92.4(5)
C(88)-C(38)-C(48)-C(58)	-0.5(7)
C(28)-C(38)-C(48)-C(58)	176.0(4)

C(38)-C(48)-C(58)-C(68)	-0.1(8)
C(48)-C(58)-C(68)-C(78)	0.7(8)
C(58)-C(68)-C(78)-C(88)	-0.7(8)
C(48)-C(38)-C(88)-C(78)	0.5(7)
C(28)-C(38)-C(88)-C(78)	-176.1(4)
C(68)-C(78)-C(88)-C(38)	0.1(8)
C(10)-C(20)-C(30)-C(30)#1	174.0(12)
C(40)-C(50)-C(60)-C(60)#1	169(4)
C(70)#2-C(70)-C(80)-C(90)	179.9(7)

Symmetry transformations used to generate equivalent atoms:

#1 -x+2,-y+1,-z+1 #2 -x+1,-y+2,-z+1

Input file for geometry optimization

```
! UKS B3LYP RIJCOSX def2-TZVP def2-TZVP/J TightSCF Grid4 NoFinalGrid
! ZORA D3BJ TightOpt NumFreq
%SCF MaxIter 500
    end
* xyzfile 0 4 3s-opt.xyz
```

Input file for calculation of hyperfine parameters

```
! UKS B3LYP RIJCOSX def2-TZVP def2-TZVP/J TightSCF Grid4 NoFinalGrid
! ZORA D3BJ
%SCF MaxIter 500
  end
%rel picturechange true end
%basis newgto C "EPRII" end
   newgto H "EPRII" end
   end
* xyzfile 0 4 3s-freq4.xyz
%eprnmr  Nuclei = all C { aiso, adip, aorb, fgrad, rho }
    Nuclei = all H { aiso, adip, aorb, fgrad, rho }
    end
```

Coordinates from geometry optimized structure

```
Fe   13.296375   16.315489    6.576215
N    11.800088   17.406037    7.314717
N    12.381610   15.826725    4.881724
C     9.508699   18.283267    7.336957
H     8.862633   17.504606    7.749093
H     8.917140   18.861367    6.628971
```

H	9.816682	18.924290	8.158472
C	10.682909	17.631319	6.644798
C	10.473440	17.241570	5.307838
H	9.549646	17.590583	4.871881
C	11.228884	16.380037	4.506280
C	10.682497	16.059035	3.133997
H	11.369543	16.425549	2.369358
H	9.711543	16.523359	2.979901
H	10.592271	14.983943	2.980107
C	11.928917	17.765211	8.687857
C	11.502436	16.865603	9.680159
C	11.736524	17.194589	11.014285
H	11.429954	16.508024	11.793567
C	12.357199	18.384376	11.359275
H	12.530531	18.629705	12.399582
C	12.773761	19.259321	10.365395
H	13.277248	20.172565	10.648086
C	12.584092	18.968839	9.017868
C	10.829941	15.548206	9.326223
H	10.699937	15.526047	8.245000
C	9.437805	15.411250	9.959410
H	9.499554	15.282729	11.042343
H	8.923392	14.538383	9.551956
H	8.823817	16.291294	9.764026
C	11.704209	14.345554	9.699724
H	12.661127	14.371759	9.178561
H	11.202452	13.414500	9.430884
H	11.906054	14.321551	10.772632
C	13.090873	19.909766	7.932512

H	13.497302	19.279390	7.136154
C	11.967477	20.760386	7.323146
H	11.242817	20.160739	6.778698
H	12.387586	21.487668	6.624975
H	11.439845	21.311887	8.105467
C	14.213179	20.833869	8.404571
H	13.836694	21.604746	9.081376
H	14.651688	21.344224	7.545435
H	15.004361	20.285814	8.907989
C	12.904639	14.707776	4.167402
C	12.507178	13.416333	4.572868
C	13.030430	12.318724	3.894717
H	12.731474	11.321120	4.187971
C	13.938715	12.477497	2.857507
H	14.335960	11.611156	2.344162
C	14.348338	13.750151	2.496439
H	15.073144	13.870476	1.701044
C	13.847945	14.879829	3.142024
C	11.550888	13.212704	5.738611
H	11.543531	14.138456	6.314816
C	10.108339	12.959209	5.278495
H	9.706450	13.810360	4.729465
H	9.462440	12.780466	6.141314
H	10.053853	12.081138	4.630628
C	12.000947	12.092925	6.680770
H	11.933919	11.113380	6.203924
H	11.358859	12.066044	7.561874
H	13.028651	12.239947	7.015241
C	14.371121	16.252327	2.765088

H	13.791970	16.989496	3.321254
C	14.213202	16.549412	1.270616
H	14.807111	15.862573	0.664930
H	14.553901	17.562564	1.044385
H	13.174697	16.454500	0.949931
C	15.840728	16.400402	3.183654
H	15.979546	16.142564	4.232703
H	16.185981	17.425069	3.028759
H	16.480576	15.738884	2.596206
C	14.839637	15.115162	7.002329
C	14.787963	16.139119	7.989482
C	15.737598	17.261823	8.072294
C	16.077746	17.786439	9.325408
H	15.597364	17.384561	10.209124
C	17.019909	18.799674	9.447640
H	17.276625	19.179824	10.429072
C	17.623916	19.340632	8.317972
H	18.348722	20.138420	8.412466
C	17.272779	18.853228	7.063653
H	17.721416	19.275795	6.173114
C	16.347798	17.826865	6.943527
H	16.086097	17.463616	5.959159
H	15.620010	15.122960	6.249486
H	14.462173	14.128560	7.248174
H	14.314815	15.895264	8.935822
